# The N-terminal region of Jaw1 has a role to inhibit the formation of organized smooth endoplasmic reticulum as an intrinsically disordered region

**DOI:** 10.1038/s41598-020-80258-5

**Published:** 2021-01-12

**Authors:** Takuma Kozono, Hiroyuki Sato, Wataru Okumura, Chifuyu Jogano, Miwa Tamura-Nakano, Yuki I. Kawamura, Jack Rohrer, Takashi Tonozuka, Atsushi Nishikawa

**Affiliations:** 1 Institute of Global Innovation Research, Tokyo University of Agriculture and Technology, Tokyo, 183-8509 Japan; 2 Department of Applied Biological Chemistry, Graduate School of Agriculture, Tokyo University of Agriculture and Technology, Tokyo, 183-8509 Japan; 3 Department of Food and Energy Systems Science, Graduate School of Bio-Applications and Systems Engineering, Tokyo University of Agriculture and Technology, Tokyo, 184-8588 Japan; 4 Communal Laboratory, Research Institute, National Center for Global Health and Medicine, Tokyo, 162-8655 Japan; 5 Department of Gastroenterology, The Research Center for Hepatitis and Immunology, Research Institute, National Center for Global Health and Medicine, Chiba, 272-8516 Japan; 6 Institute of Chemistry and Biotechnology, Zurich University of Applied Sciences, CH-8820 Waedenswil, Switzerland

**Keywords:** Organelles, Intrinsically disordered proteins, Membrane structure and assembly

## Abstract

Jaw1/LRMP is a type II integral membrane protein that is localized at the endoplasmic reticulum (ER) and outer nuclear membrane. We previously reported that a function of Jaw1 is to maintain the nuclear shape as a KASH protein via its carboxyl terminal region, a component of linker of nucleoskeleton and cytoskeleton complex in the oligomeric state. Although the oligomerization of some KASH proteins via the cytosolic regions serves to stabilize protein-protein interactions, the issue of how the oligomerization of Jaw1 is regulated is not completely understood. Therefore, we focused on three distinct regions on the cytosolic face of Jaw1: the N-terminal region, the coiled-coil domain and the stem region, in terms of oligomerization. A co-immunoprecipitation assay showed that its coiled-coil domain is a candidate for the oligomerization site. Furthermore, our data indicated that the N-terminal region prevents the aberrant oligomerization of Jaw1 as an intrinsically disordered region (IDR). Importantly, the ectopic expression of an N-terminal region deleted mutant caused the formation of organized smooth ER (OSER), structures such as nuclear karmellae and whorls, in B16F10 cells. Furthermore, this OSER interfered with the localization of the oligomer and interactors such as the type III inositol 1,4,5-triphosphate receptor (IP_3_R3) and SUN2. In summary, the N-terminal region of Jaw1 inhibits the formation of OSER as an IDR to maintain the homeostatic localization of interactors on the ER membrane.

## Introduction

The endoplasmic reticulum (ER) is a reticular membranous network consisting of a nuclear envelope (NE), sheets surrounding the nucleus and tubules in the periphery. The ER is involved in many cellular events such as protein synthesis, lipid synthesis, calcium transfer, membrane trafficking, stress responses as well as related actions. ER morphogenesis is regulated by several proteins, including Climp-63, kinectin and p180 on the sheets^1–3^ and Reticulons and DP1 on the tubules^1,4^. In addition, in the NE, a continuous region of the ER membrane, i.e. an inner nuclear membrane (INM) and an outer nuclear membrane (ONM), a linker of nucleoskeleton and cytoskeleton (LINC) complex consisting of Klarsicht/ANC-1/Syne/homology (KASH) proteins in the ONM and Sad-1/UNC84 (SUN) proteins in the INM functions as a physical linkage between cytoskeletons and nuclear lamina for the maintenance of the nuclear network such as shaping, positioning and movement^5^. To date, several proteins implicated in ER morphogenesis have been reported to be involved in hereditary spastic paraplegia (HSP) and hereditary sensory and autonomic neuropathy (HSAN), neurodegenerative diseases^6,7^. In addition, dysfunctions of nuclear physiology due to defects in the LINC complex have been reported to be causes of Emery–Dreifuss Muscular Dystrophy^8,9^, infertility^10,11^, cerebellar ataxia^12,13^ and the Hutchinson-Gilford Progeria syndrome (HGPS)^14^. It has been also reported that the expression levels of some components of the LINC complex are altered in cancer tissues^15^. Thus, the integrated morphological homeostasis of the ER including the nucleus is crucial for regulating many cellular events and physiology.

It has been reported that the overexpression of several ER resident membrane proteins results in the formation of a stacked ER with highly dense structures, such as cisternae, whorls, nuclear karmellae which are grouped as organized smooth ER (OSER)^16–25^. For example, previous investigations have reported that the ectopic expression of 3-Hydroxy-3-methyl-glutaryl-CoA (HMG-CoA) reductase^16^, the lamina-associated polypeptide 2 beta (Lap2β)^17^ starch-binding domain-containing protein 1 (Stbd1)^18^ causes the OSER formation. The oligomeric fluorescence protein tagged cytochrome b(5)^19^ and Sec61γ^19^, calnexin^20,21^ and Langerin^22^ has also been reported to form the OSER, indicating that a tightly stacked membranous environment is formed between the apposed membranes due to the irregular or excessive oligomerization of ER resident membrane proteins. In addition, OSER formation that results from the expression of mutants such as TorsinA^23^, TorsinB^24^ and the vesicle-associated membrane protein-associated protein B/C (VAPB)^25^ has also been reported to be relevant to diseases such as dystonia and amyotrophic lateral sclerosis, which suggests the existence of OSER structures under conditions of a physiological disease. Nevertheless, the details of the OSER, such as morphogenesis, plasticity and the effects on the cellular events remain to be unveiled.

Jaw1 is a type II integral membrane protein that is localized on the ER and ONM^26–28^. Current information indicates that Jaw1 is expressed in taste buds and lymphoid organs such as the spleen, thymus, bone marrow and tonsils^26,29,30^. We recently reported that Jaw1 has a role in maintaining the nuclear shape at ONM as a KASH protein^28^. Trimeric KASH proteins interact with trimeric SUN proteins in the perinuclear space (PNS) via the KASH domain and the SUN domain, respectively, resulting in the formation of a hetero-hexamer complex^31–33^. Intriguingly, some KASH proteins such as Nesprin3α and KASH5 have been reported to be oligomerized via their cytosolic regions to stabilize the interaction with proteins^27,34^. As we previously reported, Jaw1 also forms an oligomer, but the issue of whether or not the oligomerization is mediated by its cytosolic region has not been revealed^28^. Furthermore, Jaw1 has a predicted coiled-coil domain in the middle region on its cytosolic face which interacts with the type III inositol 1,4,5-triphosphate receptor (IP_3_R3), a calcium-gate channel on the ER membrane^26,30^. However, the biological significance of the interaction between these molecules is not well understood. Furthermore, the structure and function of the rest of cytosolic regions except the coiled-coil domain have also not been determined. In this study, the cytosolic face of Jaw1 was defined as follows: the N-terminal region (44-183) as an uncharacterized N-terminal region of the coiled-coil domain, the predicted coiled-coil domain (194-333), and stem region (334-469) as an uncharacterized intermediate region between coiled-coil domain and single trans-membrane domain. According to these definitions, we attempted to clarify the function of the cytosolic region of Jaw1.

The findings reported herein show that Jaw1 probably forms an oligomer via its coiled-coil domain. We also show that the N-terminal region has a role in inhibiting OSER formation, preventing the irregular oligomerization as an intrinsically disordered region (IDR), a structurally unstable region within the protein. The OSER derived from the expression of a mutant (Jaw1 ΔN) lacking the N-terminal region recruits the interactors to this structure, but does not affect the conventional ER resident proteins, calnexin and calreticulin. Finally, based on our findings, we propose that the precise oligomerization of Jaw1 regulated by the N-terminal region is crucial for the normal localization of Jaw1 at the ER and the precise localization of interactors.

## Results

### Jaw1 coiled-coil domain is a candidate for the oligomerization site

**Figure 1:**
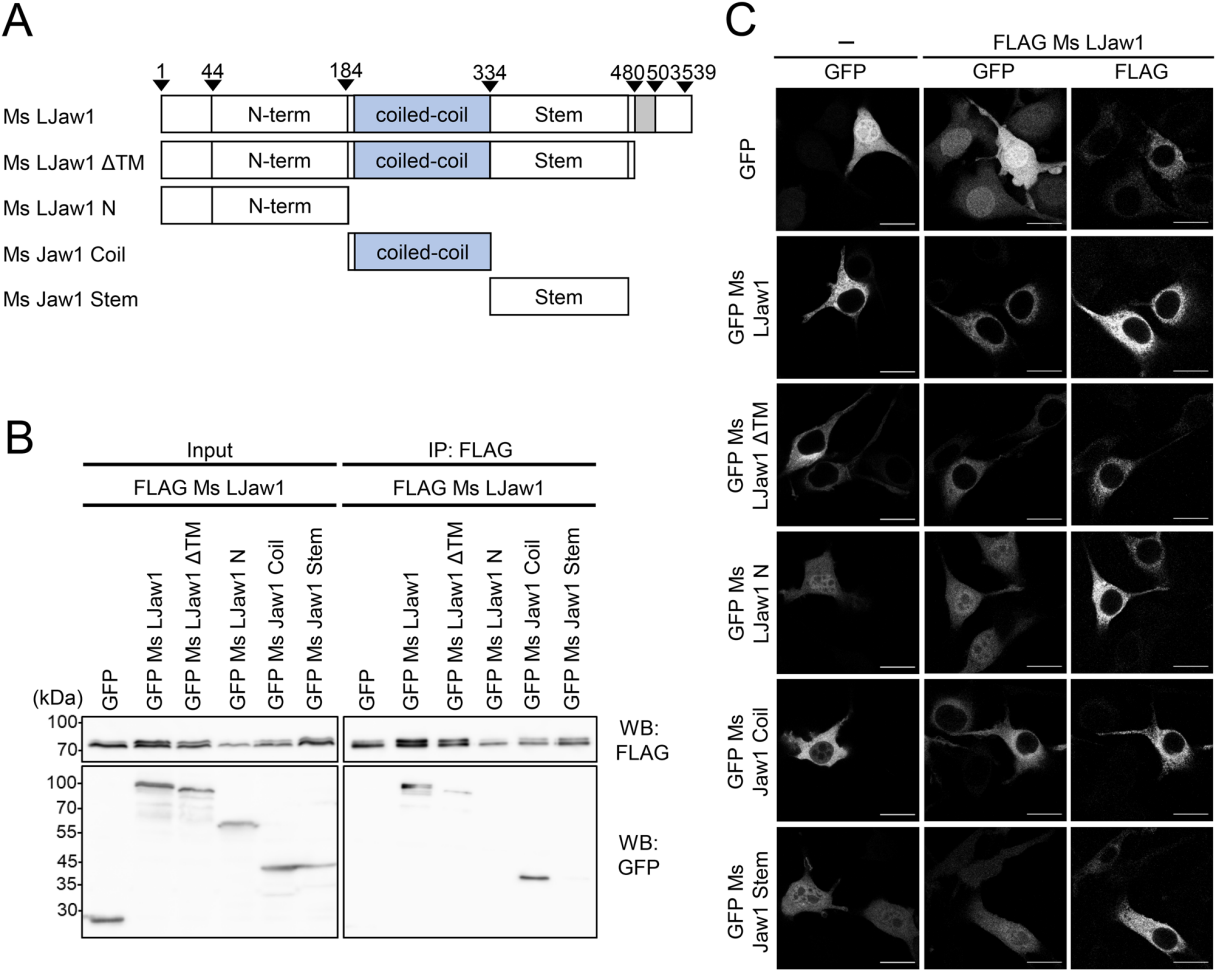
Identification of the cytosolic oligomerization site. (**A**) Schematic representation of Mouse LJaw1 (LJaw1; long form Jaw1) and mutants. N-term; N-terminal region, coiled-coil; coiled-coil domain, Stem; stem region, Gray box; single trans-membrane domain. (**B**) Identification of the oligomerization site by co-immunoprecipitation followed by western blotting. FLAG Ms LJaw1 was co-expressed with GFP, GFP Ms LJaw1 or mutants in HEK293 cells by transfection. After incubation for 24 h, the lysates were subjected to co-immunoprecipitation. For western blotting, an anti-FLAG rabbit antibody and an anti-GFP rabbit antibody as primary antibodies were used. (**C**) GFP, GFP Ms LJaw1 or mutants was expressed alone or co-expressed with FLAG Ms LJaw1 in B16F10 cells by transfection. After incubation for 24 h, the cells were subjected to immunostaining using an anti-FLAG rabbit antibody as a primary antibody. The images were acquired by confocal microscopy. Scale bar; 20 μm.

As we previously reported, Jaw1 plays a role in maintaining the nuclear shape as a KASH protein in the state of oligomer^28^. However, the oligomerization site has not been identified. Furthermore, the possibility that the oligomerization is due to the interaction between the KASH domain and the SUN domain of the PNS has not been excluded. Therefore, we first investigated the issue of whether the cytosolic region is involved in the oligomerization or not. To accomplish this, we focused on three distinct cytosolic regions: the N-terminal region of the coiled-coil domain (N-terminal region), the coiled-coil domain and the C-terminal region of the coiled-coil domain (stem region). For a co-immunoprecipitation (Co-IP) assay, we prepared plasmids encoding GFP-fused mouse Jaw1 long form (GFP Ms LJaw1) and GFP-fused mutants encoding entire cytosolic regions (GFP Ms LJaw1 ΔTM) and single regions (GFP Ms LJaw1 N, GFP Ms Jaw1 Coil or GFP Ms Jaw1 Stem) (Fig. 1A). These GFP-fused proteins were co-expressed with HA FLAG tandem tagged Ms LJaw1 (FLAG Ms LJaw1) in HEK293 cells and co-immunoprecipitated by treatment with anti-FLAG beads. The results showed that GFP Ms LJaw1 was co-immunoprecipitated by the FLAG Ms LJaw1, consistent with previous data^28^. Importantly, GFP Ms LJaw1 ΔTM and GFP Ms Jaw1 Coil were also co-immunoprecipitated by FLAG Ms LJaw1, indicating that Jaw1 forms an oligomer on the cytosolic face via the coiled-coil domain (Fig. 1B, see Supplementary Fig. S1 online).

In order to verify the oligomerization site in terms of localization, these GFP fused mutants were expressed alone or co-expressed with FLAG Ms LJaw1 in B16F10 cells derived from mouse melanoma cells followed by immunostaining. The confocal images showed that GFP Ms LJaw1, GFP Ms LJaw1 ΔTM and GFP Ms Jaw1 Coil were localized in the cytosol, whereas GFP Ms LJaw1 N and GFP Ms Jaw1 Stem were localized in the cytosol but also in the nucleus (Fig. 1C). Furthermore, the GFP Ms LJaw1 N and GFP Ms Jaw1 Stem that were localized in the nucleus were not translocated to the cytosol by the co-expression of the FLAG LJaw1, which implies that the N-terminal region and the stem region of Jaw1 do not function as oligomerization sites, consistent with Co-IP data. These data suggest that the coiled-coil domain of Jaw1 probably functions as an interaction site for the oligomerization.

### The Jaw1 N-terminal region inhibits the formation of organized smooth ER

**Figure 2:**
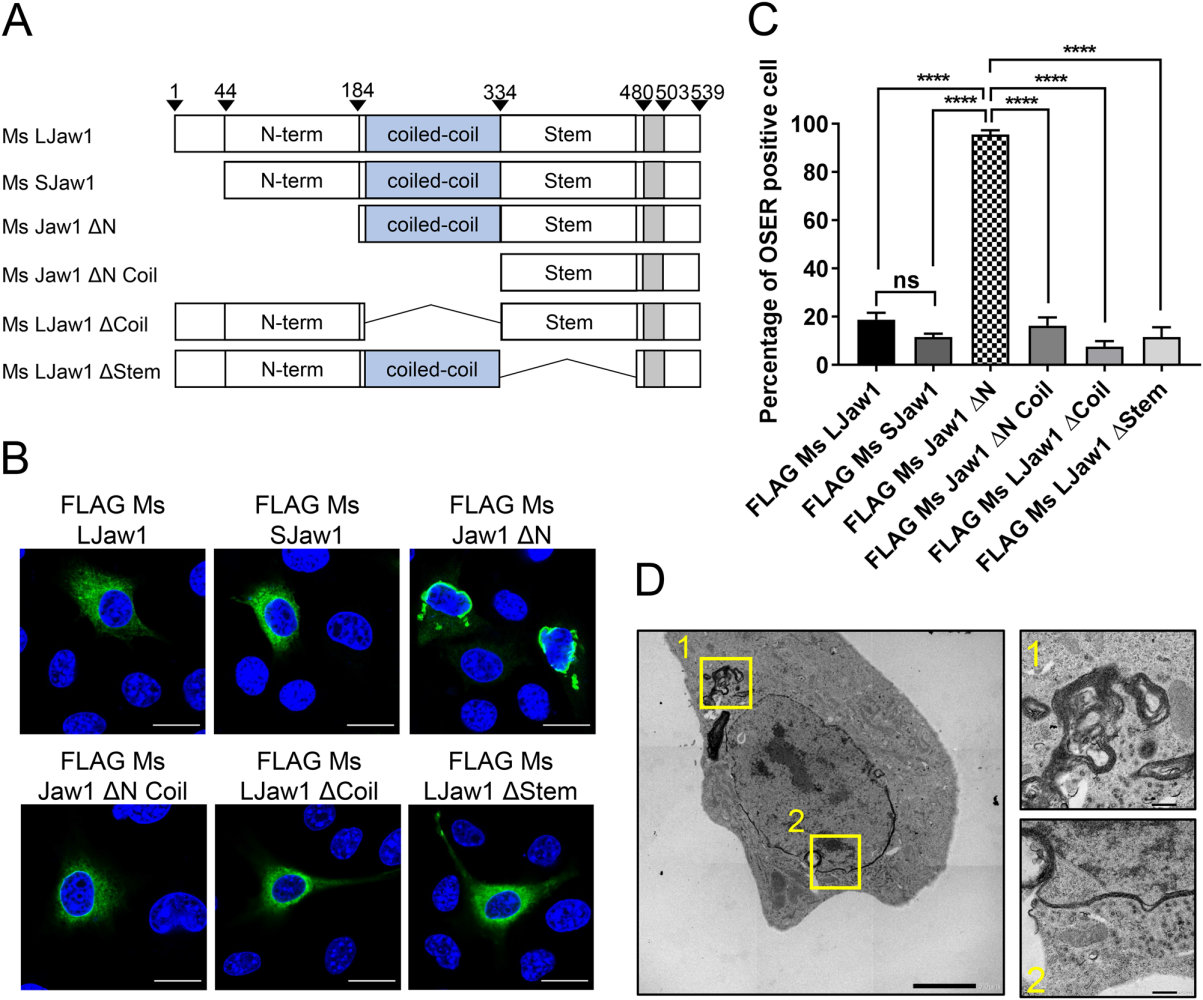
Observation of the OSER by the expression of Jaw1 ΔN. (**A**) Schematic representation of Mouse Jaw1 (LJaw1; long form Jaw1, SJaw1; short form Jaw1) and mutants lacking the N-terminal region (Ms Jaw1 ΔN), N-terminal region and coiled-coil domain (Ms Jaw1 ΔN Coil), coiled-coil domain (Ms LJaw1 ΔCoil) and stem region (Ms LJaw1 ΔStem). (**B**) FLAG Ms LJaw1, FLAG Ms SJaw1 or deletion mutants were expressed in B16F10 cells by transfection. After incubation for 24 h, immunostaining was performed using an anti-FLAG rabbit antibody (green). Nuclei were stained with Hoechst33342 (blue). The images were acquired by confocal microscopy. Scale bar; 20 μm. (**C**) Counting of the cells having OSER structures out of the FLAG positive cells in (**B**) (n = 100). In the graph, the percentage of cells with OSER structures is shown based on the average of four independent experiments per condition. Error bars show the S. D. “ns”, not significant; ****P < 0.0001, Turkey Kramer’s *t*-test. (**D**) Electron micrographs of the B16F10 cells expressing FLAG Ms Jaw1 ΔN. The magnified images are shown in the boxes at right (box 1; whorls and cisternae stacks, box 2; nuclear karmellae). Scale bar; left box; 5.0 μm, right boxes; 500 nm. The OSER positive cells were classified as the ones with a highly dense membranous structure (N = 8).

Co-IP assays showed that coiled-coil domain of Jaw1 functions as a candidate for the oligomerization site and that other cytosolic regions have no affinity for oligomerization. In order to investigate the intracellular function of each region, we prepared for several new deletion mutants of Jaw1 that lacked each region: FLAG Ms Jaw1 ΔN, FLAG Ms LJaw1 ΔCoil and FLAG Ms LJaw1 ΔStem (Fig. 2A). These mutants were expressed in B16F10 cells and the intracellular localization was then observed. Interestingly, aggregate-like structures in the cytosol and along the perinuclear membranous region were observed in nearly all of the cells that were expressing Jaw1 ΔN, a mutant lacking the N-terminal region, whereas LJaw1 and SJaw1 were localized at the conventional ER (Fig. 2B,C). These abnormal structures were observed in more detail by transmission electron microscopy. As shown in electron micrographs, these membranous structures were composed of highly dense stacked membranes, which are referred to as organized smooth ER (OSER) such as whorls (Fig. 2D, box 1) and nuclear karmellae (Fig. 2D, box 2). While whorls structure was observed in all analyzed OSER positive cells (N = 8), nuclear karmellae was observed in six out of eight cells. OSER has been reported to be caused by the ectopic overexpression of some ER resident proteins that undergo abnormal oligomerization on the ER membrane. Taking Jaw1 oligomerization via the coiled-coil domain into consideration, it was predicted that the formation of the OSER would be inhibited in cells expressing the FLAG Ms Jaw1 ΔN Coil, a mutant lacking the coiled-coil domain in addition to the N-terminal region. As expected, the percentage of cells having OSER out of the cells expressing the FLAG Ms Jaw1 ΔN Coil was compatible with FLAG Ms LJaw1 and FLAG Ms SJaw1 and much lower than that of FLAG Ms Jaw1 ΔN (Fig. 2C). This result indicates that the structural exposure of the coiled-coil domain by the deletion of the N-terminal region caused the OSER formation. In order to exclude the possibility that this OSER formation is due to the effects of the N-terminal tag, Ms Jaw1 ΔN without the tag was expressed in B16F10 cells. As a result, the cells expressing Ms Jaw1 ΔN formed significant amounts of the OSER, indicating that this OSER formation can be attributed to the deletion of the N-terminal region (see Supplementary Fig. S2A,B online). Furthermore, this OSER formation was also detected in cells that were expressing the FLAG tagged human Jaw1 ΔN (see Supplementary Fig. S3A–C online). In summary, the N-terminal region has a role in intrinsically inhibiting OSER formation in both of human and mouse Jaw1.

### The entire Jaw1 N-terminal region inhibits OSER formation

**Figure 3:**
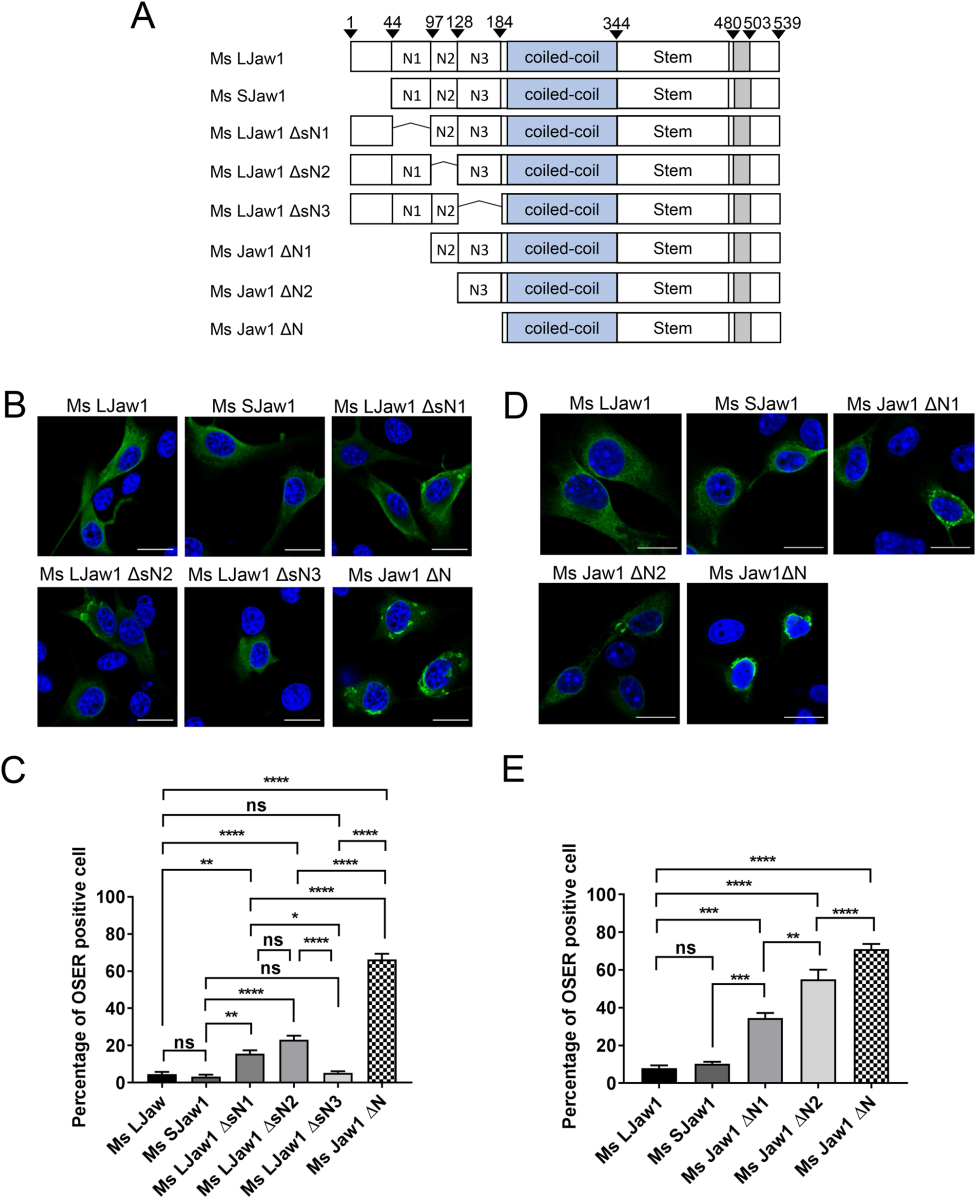
Exploration of the specific N-terminal region involved with the inhibition of OSER formation. (**A**) Schematic representation of Mouse Jaw1s or deletion mutants lacking the limited N-terminal region (Ms LJaw1 ΔsN1, Ms LJaw1 ΔsN2 and Ms LJaw1 ΔsN3) and deletion mutants with N-terminal regions of different lengths (Ms Jaw1 ΔN1, Ms Jaw1 ΔN2 and Ms Jaw1 ΔN). (**B**,**D**) Ms LJaw1, Ms SJaw1, Ms LJaw1 ΔsN1, Ms LJaw1 ΔsN2, Ms LJaw1 ΔsN3 or Ms Jaw1 ΔN (**B**) and Ms LJaw1, Ms SJaw1, Ms Jaw1 ΔN1, Ms Jaw1 ΔN2 or Ms Jaw1 ΔN (**D**) were expressed in B16F10 cells by transfection. After incubation for 24 h, immunostaining was performed using an anti-Jaw1 rat antibody (green). Nuclei were stained with Hoechst33342 (blue). The images were acquired by confocal microscopy. Scale bar; 20 μm. (**C**,**E**) Counting of the cells having OSER structures out of the Jaw1 positive cells in (**B**,**D**) (n = 100). In the graph, the percentage of cells with OSER structures is shown based on the average of four independent experiments per condition. Error bars show the S. D. “ns”, not significant; *P < 0.05; **P < 0.01; ***P < 0.001; ****P < 0.0001, Turkey Kramer’s *t*-test.

Although at least two splicing variants of Jaw1 depending on the translation initiation site, i.e. LJaw1 and SJaw1^26^, are currently known, there was no significant differences in OSER formation between them in both mouse and human Jaw1 (Fig. 2B,C, see Supplementary Figs. S2 and S3 online). These results imply that OSER formation in cells expressing Jaw1 ΔN is due to the loss of the N-terminal region (44-183). Here, in order to further investigate the issue of whether a more specific region within the N-terminal region is involved in the inhibition of OSER formation, we prepared some deletion mutants that lacked the limited N-terminal region; Ms LJaw1 ΔsN1, Ms LJaw1 ΔsN2 and Ms LJaw1 ΔsN3 and compared the extent of OSER formation with cells that were expressing Ms LJaw1, Ms SJaw1 and Ms Jaw1 ΔN (Fig. 3A). In the cells expressing Ms LJaw1 ΔsN1 and Ms LJaw1 ΔsN2, a slightly higher percentage of the OSER formation was observed compared to Ms LJaw1 and Ms SJaw1 (Fig. 3B,C). However, in the cells expressing these mutants, including Ms LJaw1 ΔsN3, the percentage of OSER formation was not as high as Ms Jaw1 ΔN. This result prompted us to consider a new hypothesis, namely, that the entire N-terminal region (44-183) of Jaw1 has a role in inhibiting OSER formation. To verify this, we prepared some new deletion mutants in which the N-terminal region had different lengths; Ms Jaw1 ΔN1 and Ms Jaw1 ΔN2 and compared the OSER formation with LJaw1, SJaw1 and Jaw1 ΔN (Fig. 3A). As expected, the percentage of OSER positive cells was high in the order; Jaw1 ΔN, Jaw1 ΔN2, Jaw1 ΔN1 and LJaw1 or SJaw1, indicating that the shorter the N-terminal region is, the higher percentage contains OSER formation (Fig. 3D, E). Thus, these data indicate that the entire N-terminal region is involved in the inhibition of OSER formation.

### Jaw1 N-terminal region inhibits its irregular oligomerization via coiled-coil domain

**Figure 4:**
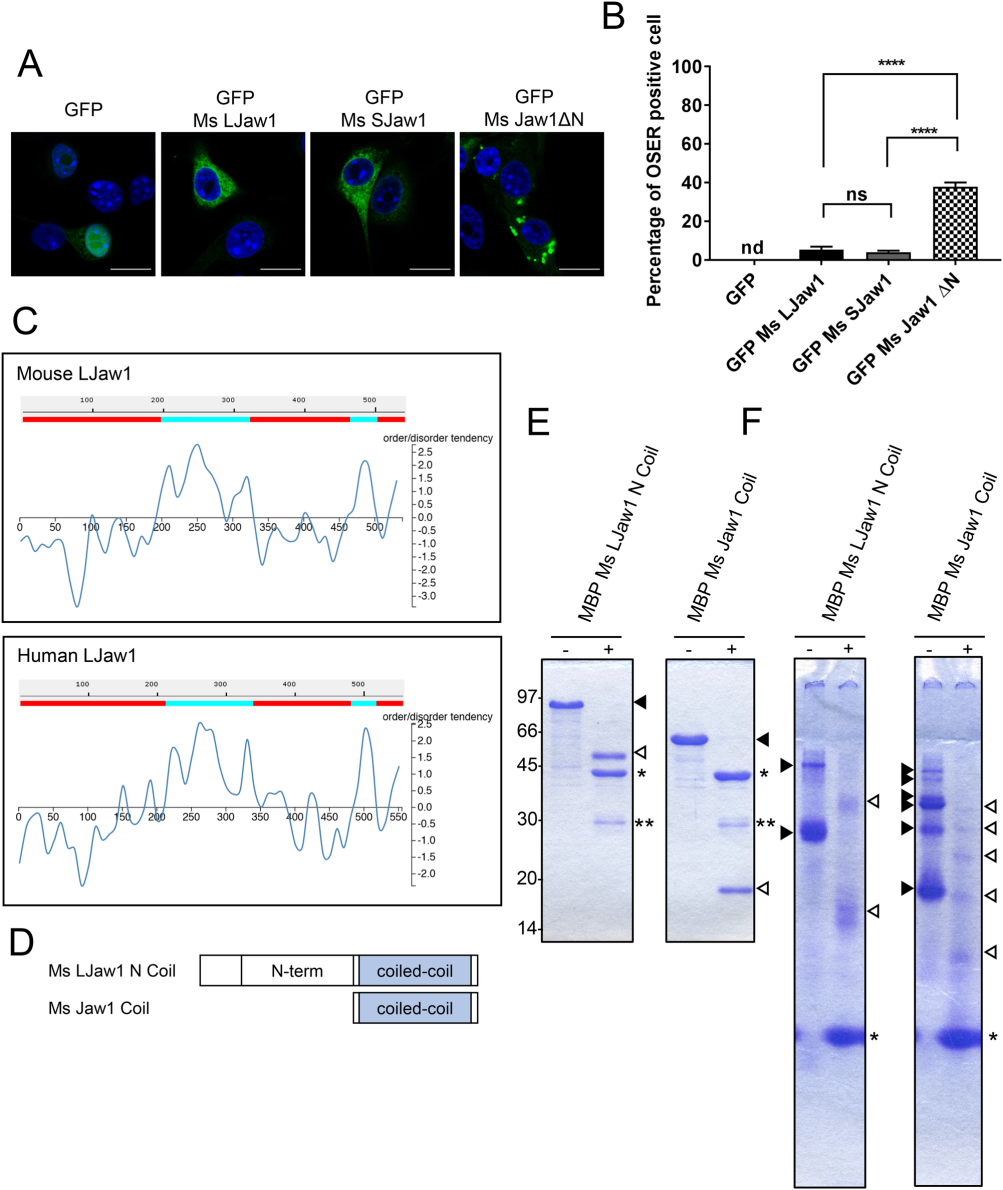
Investigation directed to which Jaw1 N-terminal region inhibits OSER formation. (**A**) GFP, GFP Ms LJaw1, GFP Ms SJaw1 or GFP Ms Jaw1 ΔN were expressed in B16F10 cells by transfection. After incubation for 24 h, the cells were fixed and incubated with PBS containing Hoechst33342 (blue). The images were acquired by confocal microscopy. Scale bar; 20 μm. (**B**) Counting of cells having OSER structures out of the GFP positive cells in (**A**) (n = 100). In the graph, the percentage of cells with OSER structures is shown based on the average of four independent experiments per condition. Error bars show the S. D. Not detectable is represented as “nd”. “ns”, not significant; ****P < 0.0001, Turkey Kramer’s *t*-test. (**C**) DICHOT analysis to search the ordered or disordered region within Jaw1. Entire amino acids sequences of Ms LJaw1 (upper) and Hu LJaw1 (bottom) were subjected to analysis by the DICHOT system. Colored areas show ordered (blue) and disordered (red) regions. The number in the side axis shows the position of the amino acids. (**D**) Schematic representation of the mutants bearing N-terminal region and coiled-coil domain (Ms LJaw1 N Coil) and coiled-coil domain alone (Ms Jaw1 Coil). (**E**,**F**) MBP Ms LJaw1 N Coil or MBP Ms Jaw1 Coil were expressed in *E*. *coli* and purified using amylose resin. The recombinant proteins treated with/without TEV protease to remove MBP tag were subjected to SDS-PAGE (**E**) and Native-PAGE (**F**) followed by CBB staining. Closed triangles, the bands of MBP tagged Jaw1; opened triangles, the bands of MBP removed Jaw1; *the band of cleaved MBP tag; **the band of TEV protease; minus, no treatment with TEV protease; plus, treatment with TEV protease.

As shown in Fig. 2B and C, in the cells expressing the FLAG Ms Jaw1 ΔN Coil, the percentage of OSER formation was significantly lower than FLAG Ms Jaw1 ΔN, compatible with FLAG Ms LJaw1 and FLAG Ms SJaw1. This result prompted us to hypothesize that the Jaw1 N-terminal region intrinsically inhibits irregular oligomerization, thus preventing the structural exposure of the coiled-coil domain. To verify this, we first investigated whether or not the addition of a GFP tag as a larger structural tag to the N-terminal of Ms Jaw1 ΔN inhibited OSER formation. The results showed that OSER formation in the cells expressing GFP Ms Jaw1 ΔN was still detected, with significant differences from GFP Ms LJaw1 and GFP Ms SJaw1 (Fig. 4A,B). These data indicate that the Jaw1 N-terminal region itself is essential for the inhibition of OSER formation. Furthermore, the amino acids sequence of mouse LJaw1 and human LJaw1 were subjected to an automated protein structure prediction: SWISS-MODEL, but the structural prediction of the regions, except for the coiled-coil domain, were not available. In order to examine the amino acids sequence further, the amino acid sequence of mouse LJaw1 and human LJaw1 were subjected to a DICHOT analysis, an established system for exploring IDR within a protein^35^. Intriguingly, the computational analysis showed, in both of human and mouse Jaw1, that the N-terminal region, the stem region and the luminal region are IDRs, whereas the coiled-coil domain and the transmembrane domain are structural regions (Fig. 4C). Furthermore, another computational analysis, D^2^P^2^ platform, also predicted that both the N-terminal region, stem region and the luminal region are IDRs (see Supplementary Fig. S4 online). To verify that N-terminal region is an IDR, the N-terminal region (Ms LJaw1 N) was produced in *E*. *coli* as an N-terminal maltose binding protein (MBP) tagged protein (MBP Ms LJaw1 N). The MBP Ms LJaw1 N was purified by affinity chromatography using an amylose resin and the MBP tag was digested with TEV protease. After the digestion, MBP and Ms LJaw1 N were separated by anion exchange chromatography and affinity chromatography using amylose resin (see Supplementary Fig. S5A online). Each recombinant protein was then subjected to circular dichroism (CD) spectroscopy. The CD spectra of Ms LJaw1 N showed the typical curve of random coil region characterized with a negative value around 200–210 nm and no distinguished change around 210–250 nm compared to that of MBP as a control of structured protein, which suggests that the N-terminal region exists as an IDR (see Supplementary Fig. S5B online)^36,37^.

Next, to investigate how the N-terminal region functions as an IDR on the oligomerization, Native-PAGE was carried out using a Ms LJaw1 N Coil bearing both the N-terminal region and the coiled-coil domain and Ms Jaw1 Coil with the coiled-coil domain alone. Here, these recombinant proteins were produced in *E*.* coli* as N-terminal MBP tagged proteins and were purified by affinity chromatography using an amylose resin (Fig. 4D). The Coomassie Brilliant Blue (CBB) staining images following SDS-PAGE showed that both the purified MBP tagged proteins were obtained in high purity (Fig. 4E, see Supplementary Fig. S5C online). Furthermore, the removal of MBP by the treatment with Tobacco Etch Virus (TEV) protease was carried out in order to exclude the structural effect of MBP. Importantly, in the CBB staining image following Native-PAGE, both the MBP Ms LJaw1 N Coil and MBP cut Ms LJaw1 N Coil appeared as mainly two bands (Fig. 4F, see Supplementary Fig. S5D online). However, both the MBP Ms Jaw1 Coil and MBP cut Ms Jaw1 Coil showed multiple bands. This result indicates that the loss of the N-terminal region changed the oligomeric states of Jaw1. In summary, these data suggest that the N-terminal region has a role in maintaining the ordered oligomerization of Jaw1 as an IDR, thus preventing the structural exposure of the coiled-coil domain, an oligomerization site. We therefore speculate that the loss of the Jaw1 N-terminal region causes the irregular oligomerization on the membrane, resulting in OSER formation in the cell. Furthermore, the result of multiple bands in the Jaw1 Coil (Fig. 4F) also implies that the coiled-coil domain is one of the direct interaction sites among Jaw1 molecules. This is consistent with the result of the co-immunoprecipitation assay in Fig. 1B.

### The OSER derived from Jaw1 ΔN separated from conventional ER network

**Figure 5:**
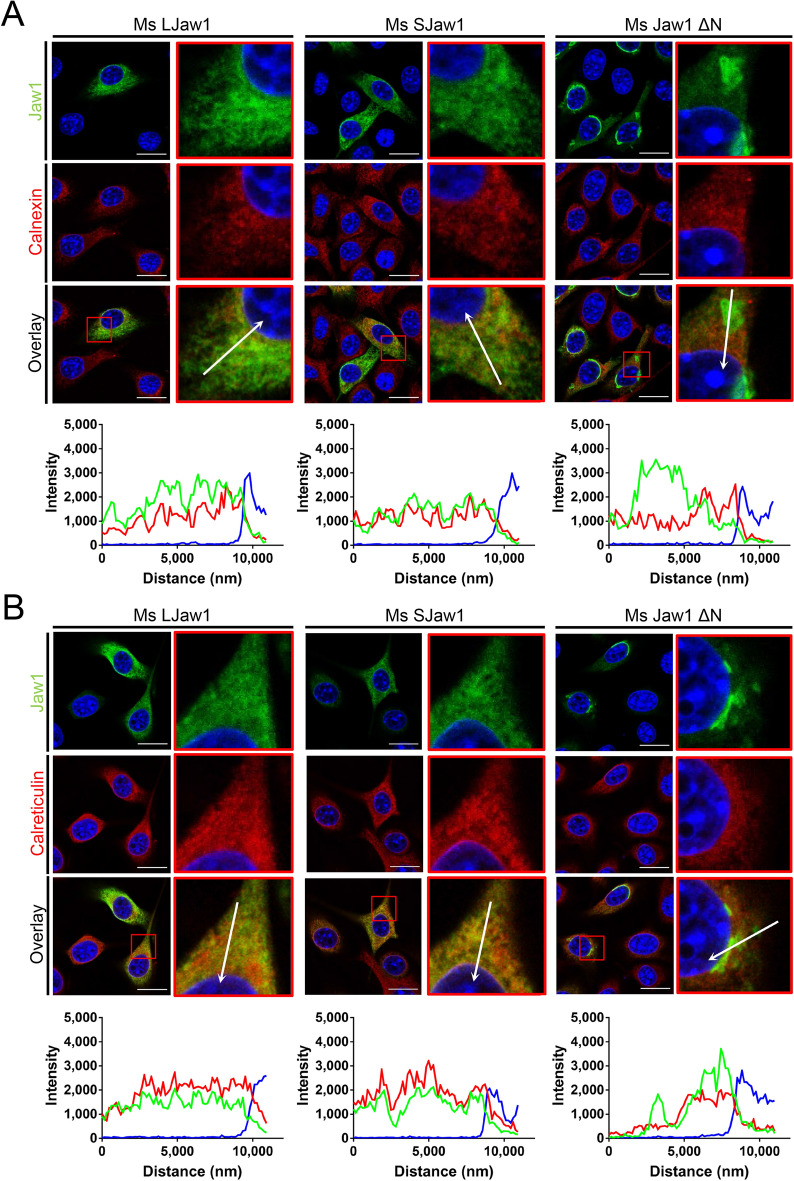
Evaluation of ER morphology under OSER formation. (**A**,**B**) Ms LJaw1, Ms SJaw1 or Ms Jaw1 ΔN were expressed in B16F10 cells by transfection. After incubation for 24 h, immunostaining was performed using an anti-Jaw1 rat antibody and an anti-calnexin rabbit antibody (**A**) or an anti-calreticulin rabbit antibody (**B**). Nuclei were stained with Hoechst33342. The images were acquired by confocal microscopy. Scale bar; 20 μm. The magnified images corresponding to the area surrounded with red lines in each image were generated and line plot profiles were performed along the arrows. Green; Jaw1, red; calnexin (**A**) or calreticulin (**B**), blue; Hoechst33342.

The effects of OSER on the cell would depend on the origins of its formation. For example, the OSER derived from the overexpression of Stbd1 recruits the interactor and changes the morphology of the ER-mitochondria network^18^. Furthermore, the OSER derived from the variant of TorsinB, an ATPases associated with diverse cellular activities, also recruits several ER-NE resident proteins such as sec61, calnexin and emerin but not protein-disulfide isomerase (PDI), LaminB1 and SUN2^24^. In order to characterize the OSER derived from Jaw1 ΔN, the effects on the localization of ER resident proteins were first evaluated. Here, in cells expressing Ms LJaw1, Ms SJaw1 or Ms Jaw1 ΔN, the localization of the ER markers; calnexin or calreticulin were observed. The confocal images and line plot profiles showed that both Ms LJaw1 and Ms SJaw1 are co-localized with calnexin nearly and calreticulin strongly within the conventional ER network (Fig. 5A,B). Furthermore, Ms Jaw1 ΔN outside the OSER was also co-localized with calnexin and calreticulin, similar to Ms LJaw1 and Ms SJaw1. However, in the compartment of OSER derived from Ms Jaw1 ΔN, calnexin and calreticulin were not recruited to the OSER at the same high level as the Ms Jaw1 ΔN. These data indicate that the OSER derived from Ms Jaw1 ΔN is likely to be a compartment that is separated from the conventional ER network and does not affect the conventional ER morphology. Furthermore, the morphology of the Golgi apparatus and mitochondria were evaluated by staining using an anti-GM130 antibody and Mito Tracker, respectively. As shown in Supplementary Fig. S6 online, the morphology and positioning were normal in the case of the OSER positive cells expressing Ms Jaw1 ΔN, the same as the cells expressing Ms LJaw1 and Ms SJaw1. Thus, the existence of OSER derived from Ms Jaw1 ΔN does not affect the morphology of these organelles.

### The OSER derived from Jaw1 ΔN caused the localization of interactors to be altered

**Figure 6:**
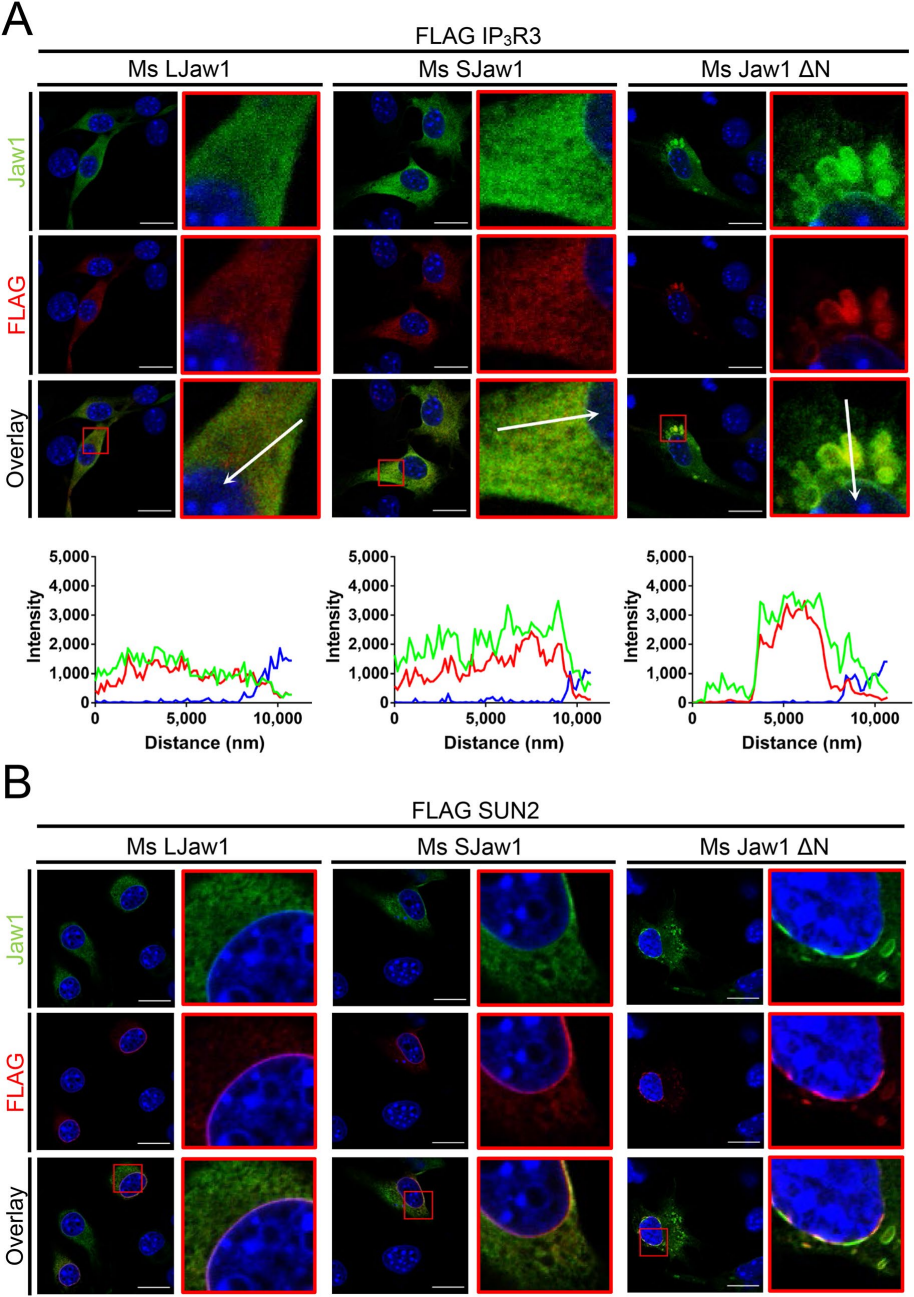
Evaluation of the effects under OSER on the localization of the interactors. **A,** (**B**) Ms LJaw1, Ms SJaw1 or Ms Jaw1 ΔN were co-expressed with FLAG IP_3_R3 (**A**) or FLAG SUN2 (**B**) in B16F10 cells by transfection. After incubation for 24 h, immunostaining was performed using an anti-Jaw1 rat antibody and an anti-FLAG rabbit antibody. Nuclei were stained with Hoechst33342. The images were acquired by confocal microscopy. Scale bar; 20 μm. The magnified images corresponding to the area surrounded with red lines in each image (**A**,**B**) were generated and line plot profiles were performed along the arrows (**A**). Green; Jaw1, red; FLAG, blue; Hoechst33342.

According to previous reports, OSER often recruits interactors to this structure^18,24^. Jaw1 has been reported to interact with IP_3_R3 localized at the ER via coiled-coil domain^30^. Furthermore, we previously reported that Jaw1 interacts with SUN proteins that are localized at the INM^28^. Therefore, we investigated the issue of whether or not these proteins are recruited to the OSER derived from Ms Jaw1 ΔN. Here, FLAG tagged IP_3_R3 (FLAG IP_3_R3) or SUN2 (FLAG SUN2) were co-expressed with Ms LJaw1, Ms SJaw1 or Ms Jaw1 ΔN in B16F10 cells and co-immunostaining using an anti-FLAG antibody and an anti-Jaw1 antibody was carried out. The confocal images and line plot profiles showed that FLAG IP_3_R3 was nearly co-localized with Jaw1 in the cells expressing Ms LJaw1 and Ms SJaw1 (Fig. 6A). Importantly, in cells expressing Ms Jaw1 ΔN, FLAG IP_3_R3 is recruited to the OSER. The counting of cells with Jaw1^+^ IP_3_R3^+^ OSER out of Jaw1^+^ cells showed that the cells expressing Ms Jaw1 ΔN formed significant amounts of OSER compared to that for Ms LJaw1 and Ms SJaw1 (see Supplementary Fig. S7 online). Furthermore, FLAG SUN2 were localized at the INM along with NE in cells expressing Ms LJaw1 and Ms SJaw1, but it was recruited to the OSER and the localization was disrupted due to the formation of nuclear karmellae in the cells expressing Ms Jaw1 ΔN (Fig. 6B). In summary, the OSER derived from Ms Jaw1 ΔN altered the localization of interactors such as IP_3_R3 and SUN2.

### The OSER derived from Jaw1 ΔN altered the localization of the oligomer

**Figure 7:**
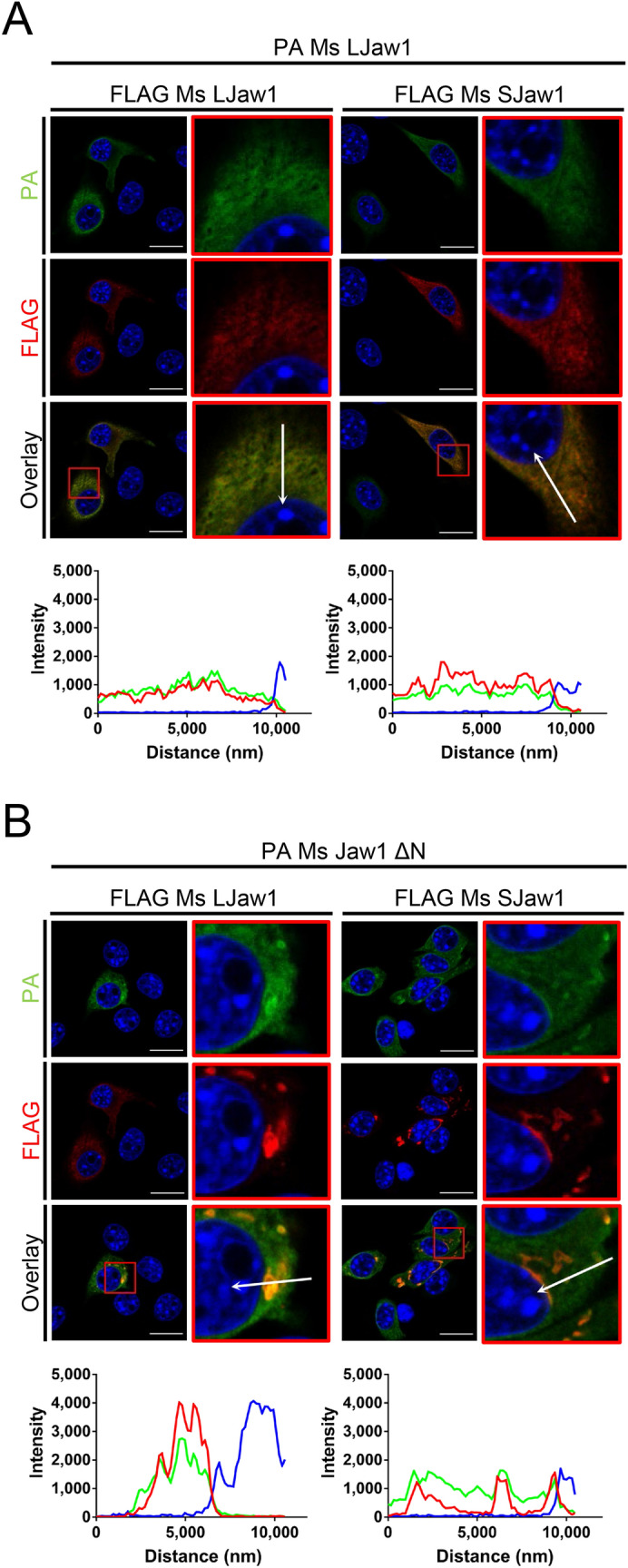
Evaluation of the effects under OSER on the localization of the oligomer. (**A**,**B**) FLAG Ms LJaw1 or FLAG Ms SJaw1 were co-expressed with PA Ms LJaw1 (**A**) or PA Ms Jaw1 ΔN (**B**) in B16F10 cells by transfection. After incubation for 24 h, immunostaining was performed using an anti-FLAG rabbit antibody and an anti-PA rat antibody. Nuclei were stained with Hoechst33342. The images were acquired by confocal microscopy. Scale bar; 20 μm. The magnified images corresponding to the area surrounded with red lines in each image were generated and line plot profiles were performed along the arrows. Green; PA, red; FLAG, blue; Hoechst 33342.

As shown in Fig. 6, the interactors are recruited to the OSER and the localization is disrupted. This led us to the hypothesis that the expression of Jaw1 ΔN also disrupts the formation and localization of the oligomer. Therefore, FLAG Ms LJaw1 or FLAG Ms SJaw1 was co-expressed with PA tagged Ms LJaw1 (PA Ms LJaw1) or Ms Jaw1 ΔN (PA Ms Jaw1 ΔN) and co-immunostaining using an anti-FLAG antibody and an anti-PA antibody was carried out. The confocal images and line plot profiles showed that FLAG Ms LJaw1 or FLAG Ms SJaw1 was almost co-localized with PA Ms LJaw1 in cells expressing these proteins (Fig. 7A). As expected, in the cells co-expressing PA Ms Jaw1 ΔN with FLAG Ms LJaw1 or FLAG Ms SJaw1, FLAG Ms LJaw1 or FLAG Ms SJaw1 were recruited to the OSER (Fig. 7B). Thus, the OSER derived from Ms Jaw1 ΔN altered the localization of the oligomer.

## Discussion

In this study, we performed functional analyses of Jaw1 cytosolic regions in terms of its oligomerization and characterized the role of the N-terminal region and coiled-coil domain. Co-immunoprecipitation experiments showed that the coiled-coil domain probably functions as an oligomerization site. On the other hand, the loss of the Jaw1 N-terminal region leads to OSER formation. According to the computational analysis using the DICHOT system and D^2^P^2^ platform and CD spectroscopy, the N-terminal region of Jaw1 exists as an IDR, and the loss of the Jaw1 N-terminal region causes irregular oligomerization in vitro, which probably results in OSER formation. Furthermore, this OSER recruits the interactors, IP_3_R3, SUN2 and full-length Jaw, but does not affect the localization of the conventional ER resident proteins, calnexin and calreticulin. In summary, these data suggest that the inhibition of OSER formation by the N-terminal region of Jaw1 as an IDR is essential for maintaining the homeostatic localization of Jaw1 oligomer and interactors on the ER membrane.

To date, it has been reported that OSER formation is frequently due to the overexpression of ER resident proteins^16–25^. Some of them are due to the structural effects by the addition of the oligomerized tags, others are derived from mutations such as TorsinB E178Q and VAPB P56S^24,25^. The OSER formation reported in this study is due to the expression of Jaw1 lacking its N-terminal region, but not the overexpression of the full-length form of Jaw1, which suggests that the function of N-terminal region is to inhibit OSER formation as an intrinsic regulator.

Furthermore, our data show that the loss of the N-terminal region caused OSER formation in both of human and mouse Jaw1. Intriguingly, a comparison of amino acid sequences between them showed that the percentage identity of the N-terminal region is relatively low (43.6%) compared to remainder of the region (71.9–92.9%) (see Supplementary Fig. S8, Table S1 online). Furthermore, a computational analysis indicated that both the N-terminal regions are IDR. The IDR is characterized by the higher polarity and lower hydrophobicity, which causes the regions to be in the unstable conformational state^38–40^. Consistently, both N-terminal regions have a lower hydrophobicity compared to structured regions; coiled-coil domain and transmembrane domain (see Supplementary Table S2 online). In addition, they also have a bigger difference between the percentage of negatively and positively charged amino acids which means there is a higher polarity compared to other regions (see Supplementary Table S2 online). Thus, these data suggest that the disorder with above features in the amino acid sequence which does not create a stable structure, in contrast to the conserved amino acids, provides the common functionality for both N-terminal regions. Furthermore, in the experiment using mutants lacking the limited N-terminal region, the percentage of OSER formation in the cells expressing Jaw1 ΔsN3 is less than Jaw1 ΔsN1 and Jaw1 ΔsN2, although all these mutants have a much lower percentage than Jaw1 ΔN. In the comparison of the amino acid composition in each N-terminal region (N1-N3), N3 region showed a relatively lower difference between the percentage of negatively and positively charged amino acids compared to N1 and N2 (see Supplementary Table S3 online). Although further wet-experiment is required for this verification, this lower polarity in N3 region would weaken the functionality as an IDR to inhibit the OSER formation compared to N1 and N2 regions. However, the most important factor for the function of the N-terminal region to inhibit the OSER formation seems to be the N-terminal length as shown in Fig. 3E.

Previous investigations reported that IDR regulates the protein-protein interaction and the intramolecular binding^38,41^. It has been also reported that IDR is involved in the regulation of precise oligomerization^42^. It is noteworthy that an in vitro assay using Jaw1 with/without the N-terminal region showed that the N-terminal region regulates the oligomeric states, which means that the irregular oligomeric state due to the lack of N-terminal region results in OSER formation. Furthermore, interestingly, the expression of Jaw1 ΔN Coil lacking N-terminal region and the coiled-coil domain repressed OSER formation. We therefore suggest that the N-terminal region of this protein has a role in inhibiting OSER formation as an IDR, thus preventing the structural exposure of coiled-coil domain, an oligomerization site. In this point, this study also supports the novel functional concept of IDR in the intrinsic regulation of oligomerization, as shown in the previous report^42^. In addition, Jaw1 ΔN, which probably causes the aberrant oligomerization, dominantly recruits the full-length Jaw1 into the OSER as shown in Fig. 7. This result perhaps indicates that the N-terminal region of full-length Jaw1 interferes with each other in its oligomer to inhibit the aberrant oligomerization at intramolecular level.

In this study, we uncovered that the coiled-coil domain is a candidate for the oligomerization site. However, we could not directly exclude the possibility that the regions except coiled-coil domain are oligomerization sites. Furthermore, a truncation mutant lacking transmembrane domain only appeared as a weak band (Fig. 1B, in the lane of co-immunoprecipitated Jaw1 ΔTM), which might mean there is a low affinity for oligomerization. We speculate the reason for this is that the oligomer consisting of Jaw1 without transmembrane domain might be unstable. Thus, we hypothesized that the insertion into the membrane is also an important factor for the precise oligomerization. On the other hand, a truncation mutant coding for only coiled-coil domain showed a strong band compatible with the full-length Jaw1 although this mutant also lacks transmembrane domain (Fig. 1B, in the lane of co-immunoprecipitated Jaw1 Coil). This would be due to the aberrant oligomerization by the loss of N-terminal region. These data indicate the possibility of additional oligomerization sites or factors for precise oligomerization, although we need further quantitative analysis.

A morphological characteristic of OSER structures derived from Jaw1 ΔN is the presence of a nuclear karmellae, where the membranes are stacked along the nuclear envelope. The reason for the expression of the Jaw1 ΔN forms this structure is probably due to the localization of Jaw1 at the ONM, as we previously reported^28^. Furthermore, this OSER recruits SUN2 and disrupts the localization at nuclear membrane in B16F10 cells, but has no dominant negative effects on the nuclear shape. In the OSER derived from Jaw1 ΔN, IP_3_R3, another interactor of Jaw1, is also recruited. IP_3_R3 releases calcium ions into the cytosol in response to an extracellular signal^43^. Furthermore, the transfer of calcium ions to mitochondria via an ER-mitochondria contact site is also involved with the regulation of apoptosis^43^. Although it has been reported that Jaw1 binds to IP_3_R3 via its coiled-coil domain, whether Jaw1 regulates the activity of IP_3_R3 remains uncovered^30^. Therefore, an evaluation of the dominant negative effects on the activity of IP_3_R3 in OSER derived from Jaw1 ΔN will be a future issue in elucidating the functional relation between Jaw1 and IP_3_R3.

In this study, we mainly focused on the cytosolic region and uncovered the physical properties of the Jaw1 N-terminal region to inhibit OSER formation. On the other hand, a few types of proteins that form OSER have been reported to be involved with certain diseases. For example, the expression of mutants such as TorsinA and VAPB are related to dystonia and amyotrophic lateral sclerosis, respectively^23,25^. According to a previous report, a mutational sequence analysis between a non-obese diabetes model (NOD/Lt) and a non-diabetic model (C57BL/6, C3H/HeJ and BALB/c) provided some candidate mutations of mouse Jaw1 that are related to type I diabetes such as G6D, L145P, duplication of three amino acids [STL (452-455)]^44^. Intriguingly, all of the mutated positions are located in the N-terminal region or stem region. Furthermore, some of the residues are also conserved in humans (see Supplementary Fig. S8 online). Taking the existence of the N-terminal region and stem region as IDRs in both of human and mouse Jaw1 as indicated by the computational analysis into consideration, these IDRs of Jaw1 would regulate the functions intrinsically. Thus, future work regarding to the issue of whether these mutations of Jaw1 affect the functions such as the ability to form oligomers and protein-protein interactions will promote the understanding how Jaw1 is involved in the pathogenesis.

In contrast to the abnormal effects leading to OSER formation, it is not clear whether the OSER is also formed under normal physiological conditions. Here, no significant difference in OSER formation between two variants (LJaw1 and SJaw1) was observed. However, interestingly, the national center for biotechnology information (NCBI) database shows the existence of a transcript variant (NM_001361633) coding for the amino acid sequence corresponding to Ms Jaw1 ΔN1, which would be a variant with a shorter N-terminal region and would form the OSER at slightly higher percentage than LJaw1 and SJaw1, as shown in Fig. 3E. Furthermore, it has been reported that the variant corresponding to Ms Jaw1 ΔN in addition to LJaw1 and SJaw1 is expressed in vitro translational system using the cDNA encoding full-length Jaw1^45^. These data suggest the possibility of OSER formation derived from an alternative splicing variant from the Jaw1 gene under normal physiological conditions. Therefore, to find tissues and cells that express this variant specifically will also provide us with an understanding of the biological significance of the OSER.

## Materials and methods

### Plasmids

The primer sets used to produce the plasmids below are shown in Supplementary Table S4 online. pcDNA5 FRT/TO HA FLAG Ms LJaw1 was produced from a total cDNA derived from mouse (C57/B6J) spleen as previously described^28^. From the same library, a DNA fragment coding Ms SJaw1 (NM_001368864.1) was amplified by PCR with primer set 1 and the PCR product was subcloned into the pcDNA5 FRT/TO HA FLAG vector (digested with *Kpn*I/*Bam*HI) using an In-Fusion HD cloning Kit (TaKaRa, Z9648), resulting in pcDNA5/FRT/TO HA FLAG Ms SJaw1. These plasmids were digested with *Kpn*I/*Bam*HI and the fragment was ligated into the pTagGFP2-C vector (Evrogen) (digested with the same enzymes), resulting in pTagGFP2-C Ms LJaw1 and SJaw1, respectively. To produce pTagGFP2-C Ms LJaw1 ΔTM coding for entire cytosolic regions, the PCR product amplified from pcDNA5FRT/TO HA FLAG Ms LJaw1 with primer set 2 was inserted into the pTagGFP2-C vector (digested with *Eco*RI/*Sal*I) using an In-Fusion HD cloning Kit. Furthermore, for the generation of the plasmids encoding Jaw1 each cytosolic region, the PCR products amplified from pTagGFP2-C Ms LJaw1 with primer sets 3, 4 and 5 were inserted into the pcDNA3.1(+) vector (Invitrogen) (digested with *Bam*HI/*Xho*I) using an In-Fusion HD cloning Kit. These plasmids were digested with *Kpn*I/*Apa*I and each fragment was then ligated into the pTagGFP2-C vector (digested with the same enzymes), resulting in pTagGFP2-C Ms LJaw1 N, Jaw1 Coil and Jaw1 Stem, respectively. For the generation of deletion mutants: pcDNA5 FRT/TO HA FLAG Ms Jaw1 ΔN and ΔN Coil, the PCR products amplified from pTagGFP2-C Ms LJaw1 using primer sets 6 and 7, respectively, were inserted into the pcDNA5/FRT/TO HA FLAG vector (digested with *Kpn*I/*Bam*HI and *Bam*HI, respectively) using an In-Fusion HD cloning Kit. Furthermore, pcDNA5 FRT/TO HA FLAG Ms Jaw1 ΔN was digested with *Kpn*I/*Bam*HI and the fragment was ligated into the pTagGFP2-C vector, resulting in pTagGFP2-C Ms Jaw1 ΔN. To produce pcDNA5 FRT/TO HA FLAG Ms LJaw1 ΔCoil and ΔStem, pcDNA5 FRT/TO HA FLAG Ms LJaw1 was performed by inverse PCR to remove the region coding for the coiled-coil domain or stem region using 5′-phosphorylated primer sets 8 and 9, respectively, and self-ligated. To remove the possible effects of the N-terminal tag, pcDNA5 FRT/TO HA FLAG Ms LJaw1 and pcDNA5 FRT/TO HA FLAG Ms SJaw1 was digested with *Kpn*I/*Bam*HI and each fragment was ligated into the pcDNA3.1(+) vector (digested with the same enzymes), resulting in the pcDNA3.1(+) Ms LJaw1 and SJaw1, respectively. In addition, for the generation of pcDNA3.1(+) Ms Jaw1 ΔN, the PCR product amplified from pTagGFP2-C Ms LJaw1 with primer set 10 was inserted into the pcDNA3.1(+) vector (digested with *Bam*HI/*Xho*I) using an In-Fusion HD cloning Kit. To produce the limited N-terminal region-truncated mutants, pcDNA3.1(+) Ms LJaw1 was performed by inverse PCR to remove the region coding for each limited N-terminal region using primer sets 11, 12 and 13, resulting into pcDNA3.1(+) Ms LJaw1 ΔsN1, ΔsN2 and ΔsN3. For the generation of pcDNA3.1(+) Ms LJaw1 ΔN1 and ΔN2, the PCR products were first amplified from pTagGFP2-C Ms LJaw1 using primer sets 14 and 15, respectively, and inserted into the pcDNA5 FRT/TO HA FLAG vector (digested with *Bam*HI/*Xho*I) using an In-Fusion HD cloning Kit. These plasmids were then digested with *Bam*HI/*Xho*I and each fragment was ligated into the pcDNA3.1(+) vector (digested with the same enzymes). To generate pcDNA3.1(+) PA Ms LJaw1 and Jaw1 ΔN, pcDNA3.1(+) PA vector was first prepared by the insertion of DNA cassette encoding PA tag (primer set 16) into the pcDNA3.1(+) vector (digested with *Nhe*I/*Hind*III). pcDNA3.1(+) Ms LJaw1 and Jaw1 ΔN were then digested with *Kpn*I/*Bam*HI and *Kpn*I/*Xho*I, respectively, and each fragment was ligated into the pcDNA3.1(+) PA vector. To produce the pMALcRI TEV Ms LJaw1 N, LJaw1 N Coil and the Jaw1 Coil, the PCR products amplified from pTagGFP2-C Ms LJaw1 with primer sets 3, 17 and 4, respectively, were inserted into the pcDNA3.1(+) vector (digested with *Bam*HI/*Xho*I) using an In-Fusion HD cloning Kit. These plasmids were digested with *Bam*HI/*Xho*I and each fragment was then ligated into the pMALcRI TEV vector (previously described^28^) (digested with *Bam*HI/*Sal*I), resulting in pMALcRI TEV Ms LJaw1 N, LJaw1 N Coil, Jaw1 Coil, respectively. For the generation of the pcDNA5 FRT/TO HA FLAG Hu LJaw1, SJaw1 and Jaw1 ΔN, the PCR products were amplified from a total cDNA derived from Burkitt’s lymphoma cell line (Raji) with primer sets 18, 19 and 20 and inserted into the pcDNA3.1 (+) vector (digested with *Bam*HI/*Xho*I) using an In-Fusion HD cloning Kit. pcDNA5 FRT/TO HA FLAG Ms SUN2 was produced from EGFP-C1 SUN2 (kindly provided by Howard, J. Worman^46^) as previously described^28^. pCMV6 Myc-DDK-tagged Mouse IP_3_R3 (NM_08053) was purchased from Ori Gene (MR225699). The identity of cloned sequences was verified by the DNA sequence contract service (eurofin). After confirmation, each plasmid was prepared using the Plasmid Mini Kit (Qiagen, 12123) and used for following experiments.

### Cells and cell culture

B16F10 and HEK293 cells were incubated in MEM supplemented with 10% fetal bovine serum (FBS) and 5.84 mg/mL of _L_-glutamine and in DMEM containing 10% FBS, 100 U/mL penicillin, 100 μg/mL streptomycin (Sigma) and 5.84 mg/mL l-glutamine, respectively, and maintained at 37 °C in 5% CO2.

### Transfection

Plasmids were introduced into B16F10 or HEK293 cells using Screen*Fect*^TM^A *plus* (FUJIFILM Wako Pure Chemical, 299-77103) according to the manufacturer’s instructions. After the treatment for 24 h, the cells were used for the following assays.

### Immunostaining

B16F10 cells were grown on 8 well chamber slides (SARSTEDT, 94.6032.039). After the transfection, the cells were washed with PBS and fixed in 4% paraformaldehyde (PFA)/PBS for 10 min. After washing with PBS, the cells were permeabilized by treatment with 0.1% Triton X-100/PBS for 5 min. After washing with PBS, the cells were blocked with 3% bovine serum albumin (BSA) diluted with PBS for 1 h and reacted with the following primary antibodies diluted with 1% BSA/PBS for 1 h; anti-FLAG mouse monoclonal antibody (1:500) (Sigma, F1804), anti-FLAG rabbit antibody (1:500) (CST, D6W5B), anti-PA rat antibody (1:500) (FUJIFILM Wako Pure Chemical Corporation, 016-25861), anti-GM130 mouse antibody (1:250) (BD Transduction Laboratories™, 610822), anti-calnexin rabbit antibody (1:500) (Merck Millipore, AB2301), anti-Calreticulin rabbit antibody (1:200) (ENZO Life Science, ADI-SPA-600) and anti-Jaw1 rat antibody (1:200) (produced in our laboratory as previously described (28)). After washing with 0.1% BSA/PBS for 5 min three times, the cells were incubated with the following secondary antibodies diluted into 1:500 with 0.1% BSA/PBS containing Hoechst33342 for 1 h; Alexa Fluor™ 488 goat anti-mouse IgG (H + L) (Thermo Fisher Scientific, A11001), Alexa Fluor™ 488 goat anti-rabbit IgG (H + L) (Thermo Fisher Scientific, A11008), Alexa Fluor™ 488 goat anti-rat IgG (H + L) (Thermo Fisher Scientific, A11006), Alexa Fluor™ 568 goat anti-mouse IgG (H + L) (Thermo Fisher Scientific, A11004) and Alexa Fluor™ 568 goat anti-rabbit IgG (H + L) (Thermo Fisher Scientific, A11011). After washing with 0.1% BSA/PBS for 5 min three times, the cells were mounted with VECTOR SHIELD (Vector laboratories). For the staining of mitochondria, Mito Tracker Orange CMTMRos (Thermo Fisher Scientific, M7510) was used before the fixation according to manufacturer’s instructions. The images for statistics were acquired using fluorescence microscopy (Leica, AF6000-DMI6B) (Objective lens; HCX PL FLUOTAR 63×/1.25 OIL PH3). The confocal images were acquired using confocal microscopy (Zeiss, LSM710) (Objective lens; Zeiss Plan Apo-chromat 63 × 1.4 NA).

### Co-immunoprecipitation

HEK293 Cells were cultured on a 6 well plate (TrueLine, TR5000). After the transfection, the cells were peeled off by gentle pipetting and centrifuged at 500×*g* for 10 min at 4 °C. The pellets were lysed in lysis buffer (50 mM Tris–HCl pH 7.6, 150 mM NaCl, 0.5% NP-40) containing 1 µL of a protease inhibitor cocktail (Nacalai Tasque, 04080-24). The lysates were sonicated for 10 min on ice and centrifuged at 12,000×*g* for 20 min at 4 °C. A portion of the supernatants was used as an input and the remainder was added to Anti DYDDDDK tag Antibody Beads (FUJIFILM Wako Pure Chemical Corporation, 012-22781) equilibrated with incubation buffer (50 mM Tris–HCl pH 7.6, 150 mM NaCl, 0.1% NP-40) at 4 °C for 2 h. The beads were then collected by brief centrifugation and washed four times with washing buffer (50 mM Tris–HCl pH 7.6, 150 mM NaCl). For elution, the beads were mixed with SDS-PAGE buffer and heat blocked at 95 °C for 5 min. After a brief centrifugation, the supernatants were subjected to western blotting.

### Western blotting

The samples were subjected to western blotting following SDS-PAGE. Polyvinylidene Fluoride membranes were blocked in 3% skim milk (FUJIFILM Wako Pure Chemical Corporation, 190-12865) diluted with Tris-Buffered Saline (TBS) (20 mM Tris–HCl pH7.6 and 137 mM NaCl) containing 0.1% Tween-20 (TBS-T) for 1 h. After washing the membrane with TBS-T, it was reacted with the following primary antibodies diluted with 1% skim milk/TBS-T for overnight; anti-FLAG rabbit antibody (1:1000) (CST, D6W5B) and anti-GFP rabbit polyclonal antibody (1:1000) (Thermo Fisher Scientific, A-11122). After washing the membrane with TBS-T, it was incubated with a secondary antibody (ECL™ Anti-rabbit IgG, HRP-Linked Whole Antibody Sheep (GE Healthcare, NA934)) diluted into 1:5000 with TBS-T for 1 h. After washing the membrane with TBS-T, Immunostar Zeta (FUJIFILM Wako Pure Chemical Corporation, 291-72401) was used as substrates and the bands were detected using LAS3000.

### Transmission electron microscopy

For observing OSER structures using transmission electron microscopy, the samples were prepared as previously described^47^. B16F10 cells grown in a 35 mm dish and transfected with pcDNA5 FRT/TO HA FLAG Ms Jaw1 ΔN were prefixed by treatment with 2% PFA and 2% glutaraldehyde in 30 mM HEPES buffer (pH 7.4) for 4 h at room temperature. After washing with 30 mM HEPES containing 5% sucrose gently twice, post-fixation was performed in a 1% OsO_4_ mixture containing 0.8% K_3_[Fe(CN)_6_] in 30 mM HEPES buffer (pH 7.4) for 1 h at room temperature. After washing with hyperpure water, the cells were stained en bloc with EM stainer (Nisshin EM) and dehydrated in ethanol, and then embedded in Quetol 812 (Nisshin EM). The blocks were sectioned using an ultramicrotome (Leica EM UC7; Leica) and were then observed by electron microscopy (JEM-1400; JEOL).

### Analysis of recombinant proteins

BL21(DE3) transformed with pMALcRI TEV Ms LJaw1 N Coil and Ms Jaw1 Coil were cultured in three test tubes containing 3 mL LB medium and the expression of the MBP Ms LJaw1 N Coil or the Ms Jaw1 Coil were induced by treatment with 1 mM isopropyl β-d-1-thiogalactopyranoside for 4 h at 25 °C. 9 mL of culture media was centrifuged at 8000 rpm for 10 min and the pellets were resuspended in washing buffer (10 mM Tris–HCl pH 7.4 and 200 mM NaCl). The suspensions were sonicated on ice and centrifuged at 12,000 rpm for 30 min. The supernatants were performed for purification using an amylose resin (New England Biolabs, E8021). MBP tag was removed by the treatment of TEV proteases (0.58 mg/mL) for 16 h at 4 °C. The purified recombinant proteins were mixed with SDS-PAGE sample buffer, heat blocked at 95 °C for 5 min and subjected to SDS-PAGE. The resulting purified proteins were mixed with Native-PAGE sample buffer and subjected to Native-PAGE. The gels were then subjected to CBB staining. For the preparation of the samples for the circular dichroism spectrometry, BL21(DE3) transformed with pMALcRI TEV Ms LJaw1 N was cultured in 1 L LB medium and the expression of the MBP Ms LJaw1 N was induced by treatment with 1 mM isopropyl β-d-1-thiogalactopyranoside for 4 h at 25 °C. The culture media was centrifuged at 8000 rpm for 10 min and the pellet was resuspended in washing buffer. The suspension was sonicated on ice and centrifuged at 12,000 rpm for 30 min. The supernatant was purified using an amylose resin. Purified MBP Ms LJaw1 N was treated with TEV proteases (0.58 mg/mL) for 16 h at 30 °C to remove the MBP tag, resulting to MBP tag and Ms LJaw1 N. After digestion, the His tagged TEV proteases was removed by the affinity chromatography using Ni–NTA Agarose (Qiagen, 1018244). The solution containing MBP tag and Ms LJaw1 N was concentrated with Amicon Ultra-15 Centrifugal Filter (Millipore, UFC901008), desalted in 20 mM Tris–HCl pH 8.0 and each protein was separated by anion exchange chromatography [HiTrap Q FF (GE Healthcare, 17515601)] on FPLC ÄKTAPurifier system (GE Healthcare). A gradient of 0 to 50% of buffer B (20 mM Tris–HCl pH 8.0 and 1 M NaCl) was applied. The fractions containing Ms LJaw1 N were concentrated with Amicon Ultra-15 Centrifugal Filter, desalted in 20 mM Tris–HCl pH 8.0 and subjected to anion exchange chromatography followed by affinity chromatography using amylose resin to remove the MBP tag or MBP tagged proteins. Each solution containing MBP or Ms LJaw1 N was concentrated with Amicon Ultra-15 Centrifugal Filter and desalted in 20 mM Tris–HCl pH 8.0. After that, the samples were mixed with SDS-PAGE sample buffer, heat blocked at 95 °C for 5 min and subjected to SDS-PAGE followed by CBB staining.

### Circular dichroism spectroscopy

CD spectroscopy was performed as previously described^48^. Briefly, purified MBP or Ms LJaw1 N were dissolved in 20 mM Tris–HCl pH7.4, 200 mM NaCl at a concentration of 0.1 mg/mL. CD spectra were recorded on a J-720WI spectrophotometer (Jasco GmbH) using at a 0.1 cm optical path, 0.1 nm interval, 1.0 nm bandwidth and 50 nm/min scanning speed. The spectra were recorded five times followed by averaging and background subtraction. The HT voltage was below 700 V over the entire range of recording (200–250 nm).

### Computational exploration of intrinsically disordered region

The DICHOT algorithm and D^2^P^2^ platform to explore the intrinsically disordered region within the amino acid sequence coding for Jaw1 were used in the online page below (http://210.137.243.169/dichot/) and (http://d2p2.pro), respectively^35,49–57^. The entire amino acid sequences of human and mouse Jaw1 were analyzed.

### Statistical analysis

The collected data were analyzed and graphically presented using GraphPad Prism7 (GraphPad). Statistical significance was determined by one-way ANOVA followed by Tukey Kramer’s *t*-test.

## Supplementary Information

Supplementary Information.

Supplementary Table S4.

## Data Availability

The datasets generated during and/or analyzed during the current study are available from the corresponding author on reasonable request.
