# Supplementary material for: The N-terminal region of Jaw1 has a role to inhibit the formation of organized smooth endoplasmic reticulum as an intrinsically disordered region
Source: Sci Rep. 2021 Jan 12;11:753. doi: 10.1038/s41598-020-80258-5 (PMC7804115; doi:10.1038/s41598-020-80258-5)
Supplement: Supplementary file 1 — Supplementary Information. [file 41598_2020_80258_MOESM1_ESM.pdf]

Supplementary Information for:

The N-terminal region of Jaw1 has a role to inhibit the formation of organized smooth endoplasmic reticulum as an intrinsically disordered region

Takuma Kozono[1, 5], Hiroyuki Sato[2], Wataru Okumura[3], Chifuyu Jogano[2], Miwa Tamura-Nakano[4], Yuki I. Kawamura[5], Jack Rohrer[6], Takashi Tonozuka[2], Atsushi Nishikawa[1,2,3*]

[1]Institute of Global Innovation Research, Tokyo University of Agriculture and Technology, Tokyo, 183-8509, Japan.
[2]Department of Applied Biological Chemistry, Graduate School of Agriculture, Tokyo University of Agriculture and Technology, Tokyo, 183-8509, Japan.
[3]Department of Food and Energy Systems Science, Graduate School of Bio-Applications Systems Engineering, Tokyo University of Agriculture and Technology, Tokyo, 183-8509, Japan.
[4]Communal Laboratory, Research Institute, National Center for Global Health and Medicine, Tokyo, 162-8655, Japan.
[5]Department of Gastroenterology, The Research Center for Hepatitis and Immunology, Research Institute, National Center for Global Health and Medicine, Chiba, 272-8516, Japan.
[6]Institute of Chemistry and Biotechnology, Zurich University of Applied Sciences, Waedenswil, CH-8820, Switzerland.

*corresponding. nishikaw@cc.tuat.ac.jp

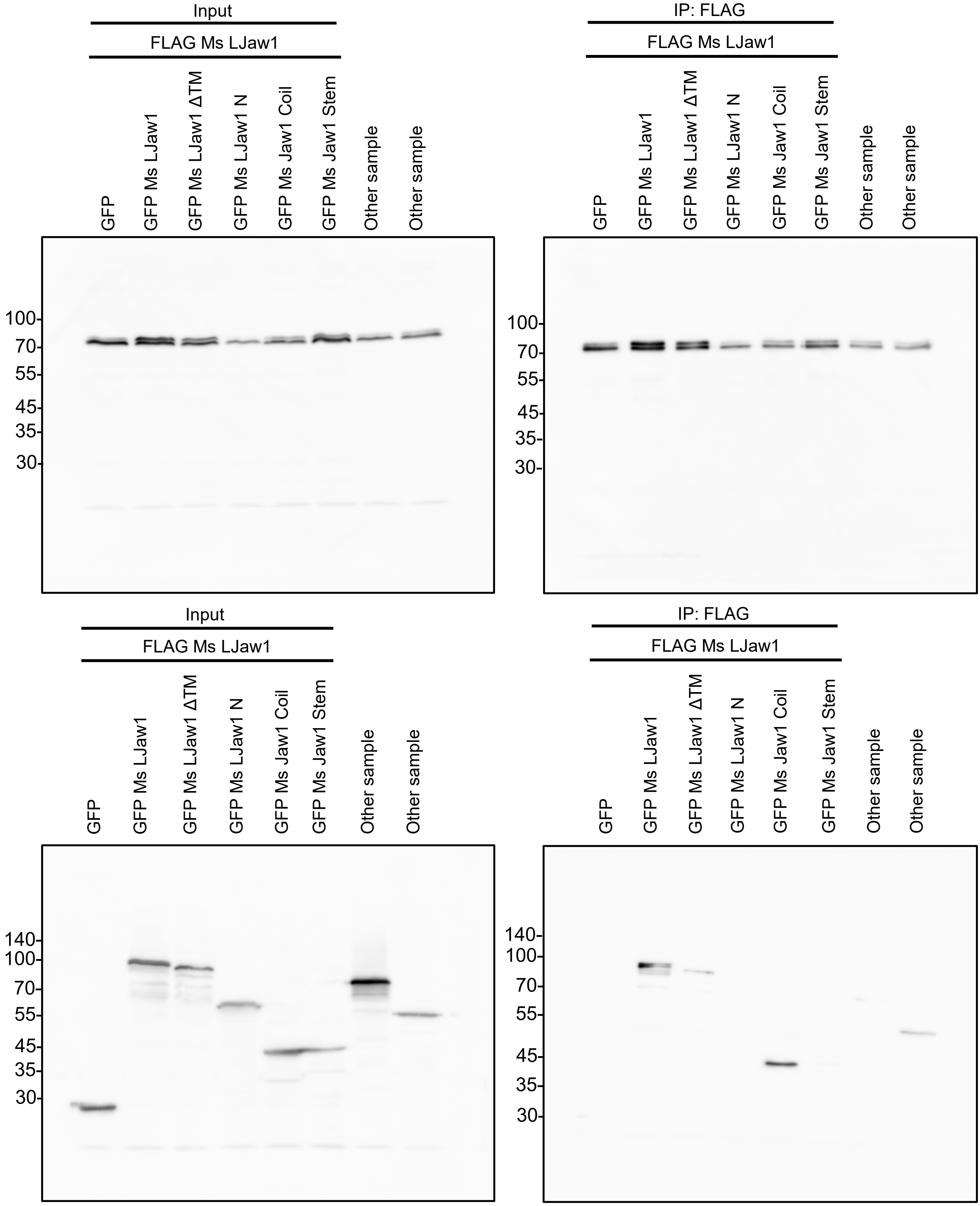

Supplementary Figure S1. Identification of the oligomerization site by co-immunoprecipitation followed by western blotting. The full-length blots of Fig. 1B. For western blotting, an anti-FLAG rabbit antibody (upper images) and an anti-GFP rabbit antibody (bottom ones) as primary antibodies were used. The lanes of "Other sample" are not related to this study.

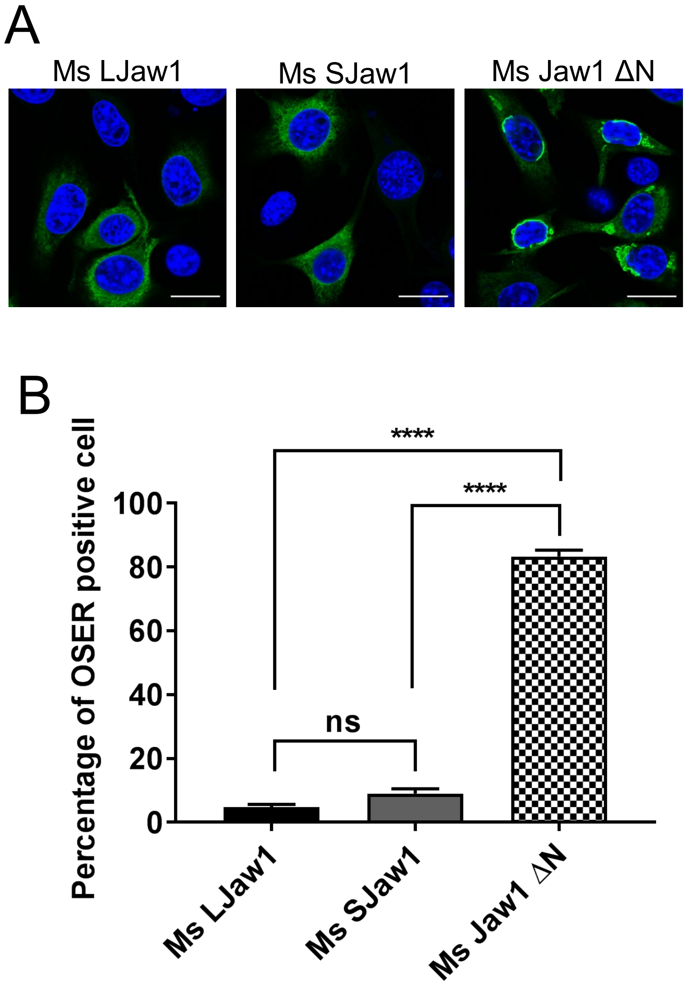

Supplementary Figure S2. Observation of the OSER by the expression of Jaw1 ΔN without tag. **A)** Ms LJaw1, Ms SJaw1 or Ms Jaw1 ΔN were expressed in B16F10 cells by transfection. After incubation for 24 h, immunostaining was performed using an anti-Jaw1 rat antibody as a primary antibody and an Alexa Fluor 488-labelled goat anti-rat IgG antibody as a secondary antibody. Nuclei were stained with Hoechst33342 (blue). The images were acquired by confocal microscopy. Scale bar; 20 μm. **B**) Counting of the cells having OSER structures out of the Jaw1 positive cells in (A) (n =100). In the graph, the percentage of cells with OSER structures is shown based on the average of four independent experiments per condition. Error bars show the S. D. "ns", not significant; ****, $P < 0.0001$, Turkey Kramer's *t*-test.

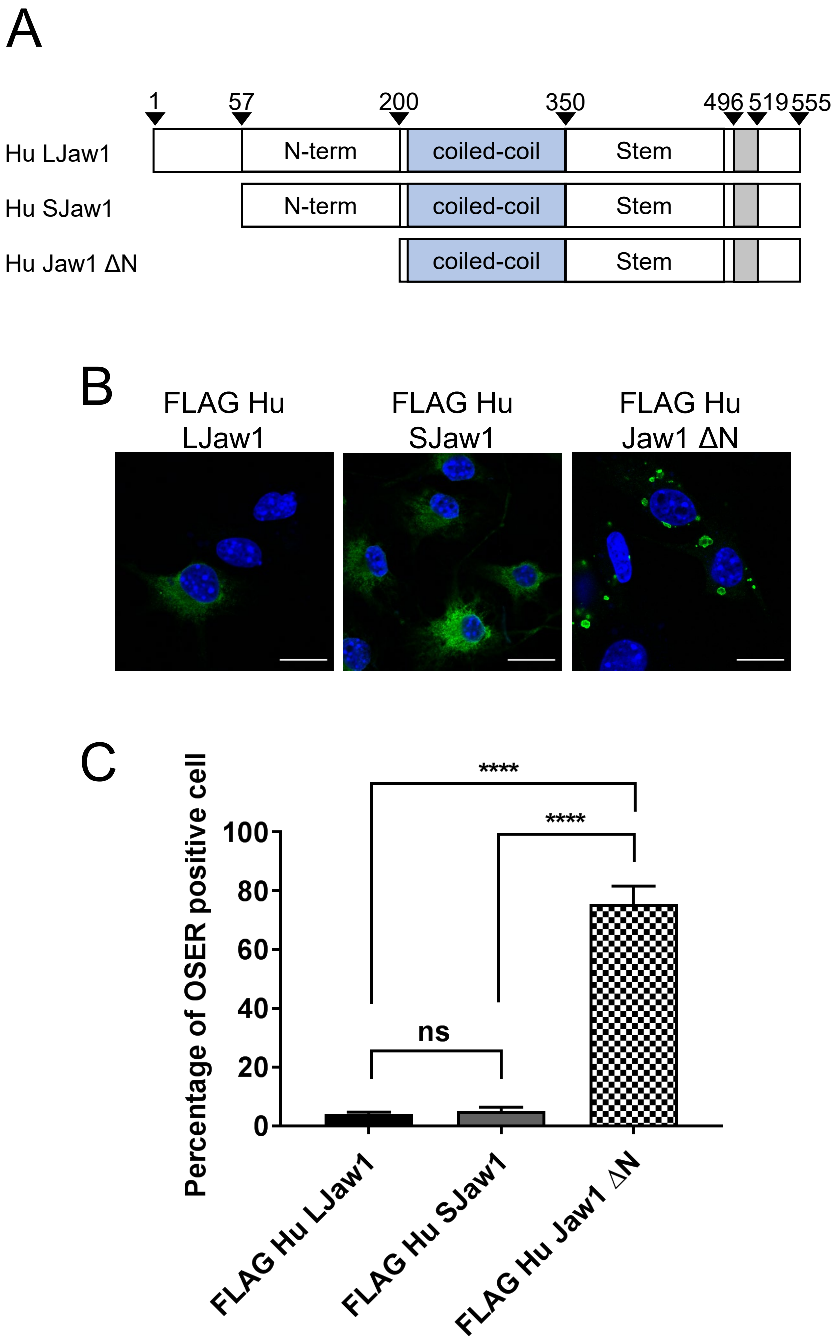

Supplementary Figure S3. Observation of the OSER by the expression of human Jaw1 ΔN. **A)** Schematic representation of human Jaw1 (LJaw1; long form Jaw1, SJaw1; short form Jaw1) and a mutant lacking the N-terminal region (Hu Jaw1 ΔN) **B)** FLAG Hu LJaw1, FLAG Hu SJaw1 or FLAG Hu Jaw1 ΔN were expressed in B16F10 cells by transfection. After incubation for 24 h, immunostaining was performed using an anti-FLAG rabbit antibody as a primary antibody and an Alexa Fluor 488-labelled goat anti-rabbit IgG antibody as a secondary antibody. Nuclei were stained with Hoechst33342 (blue). The images were acquired by confocal microscopy. Scale bar; 20 μm. **C**) Counting of the cells having OSER structures out of the FLAG positive cells in (B) (n =100). In the graph, the percentage of cells with OSER structures is shown based on the average of four independent experiments per condition. Error bars show the S. D. "ns", not significant; ****, $P < 0.0001$, Turkey Kramer's *t*-test.

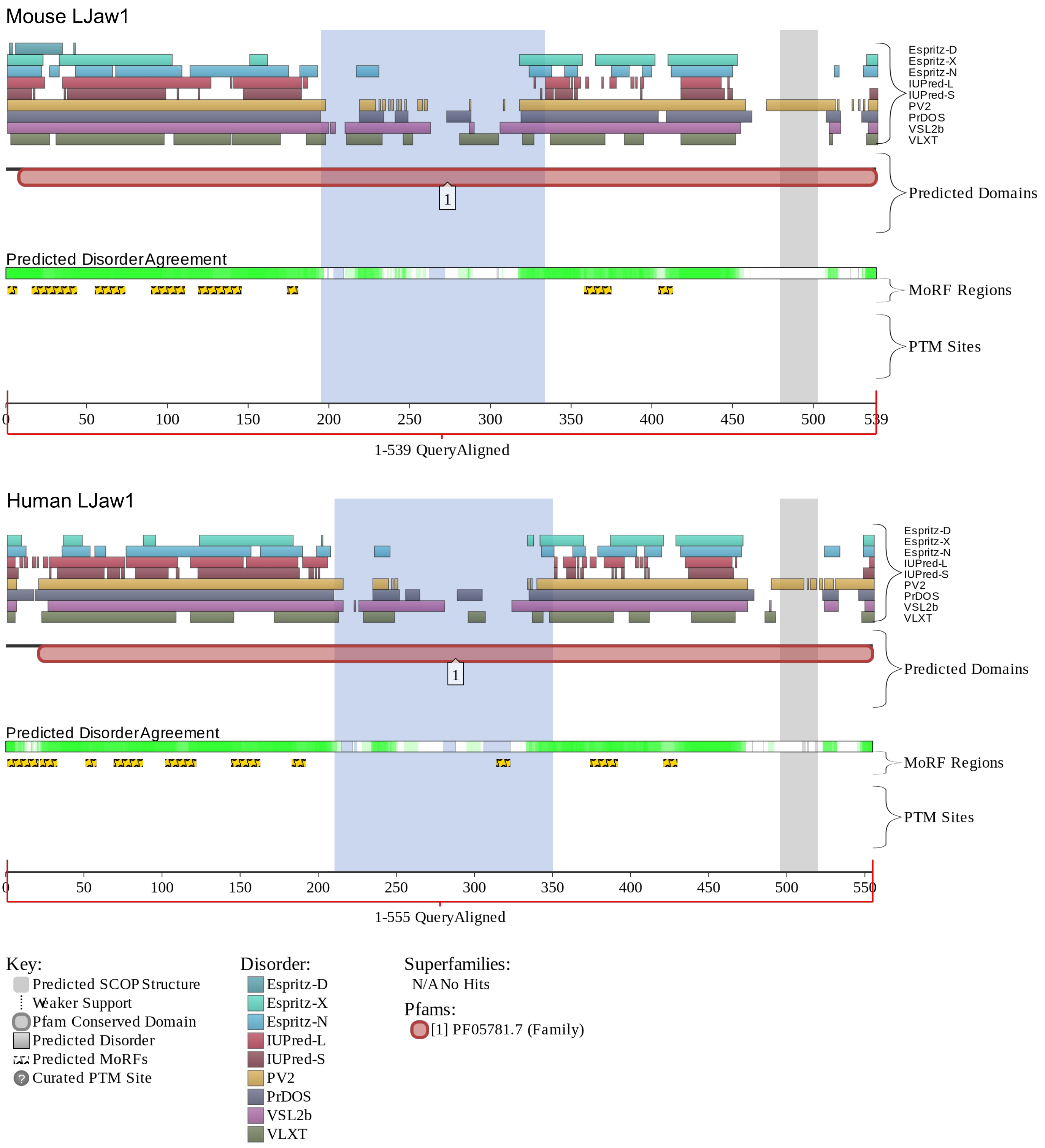

Supplementary Figure S4. Exploration of IDR within Jaw1 by D^2P^2 platform. Entire amino acids sequences of Ms LJaw1 (upper) and Hu LJaw1 (bottom) were subjected to analysis. Transparent colored areas in the back show coiled-coil domain (blue) and single trans-membrane domain (gray). Each colored box corresponds to the IDR positions predicted by each algorithm. The axis in the bottom shows the length and position of the amino acids comprising each Jaw1.

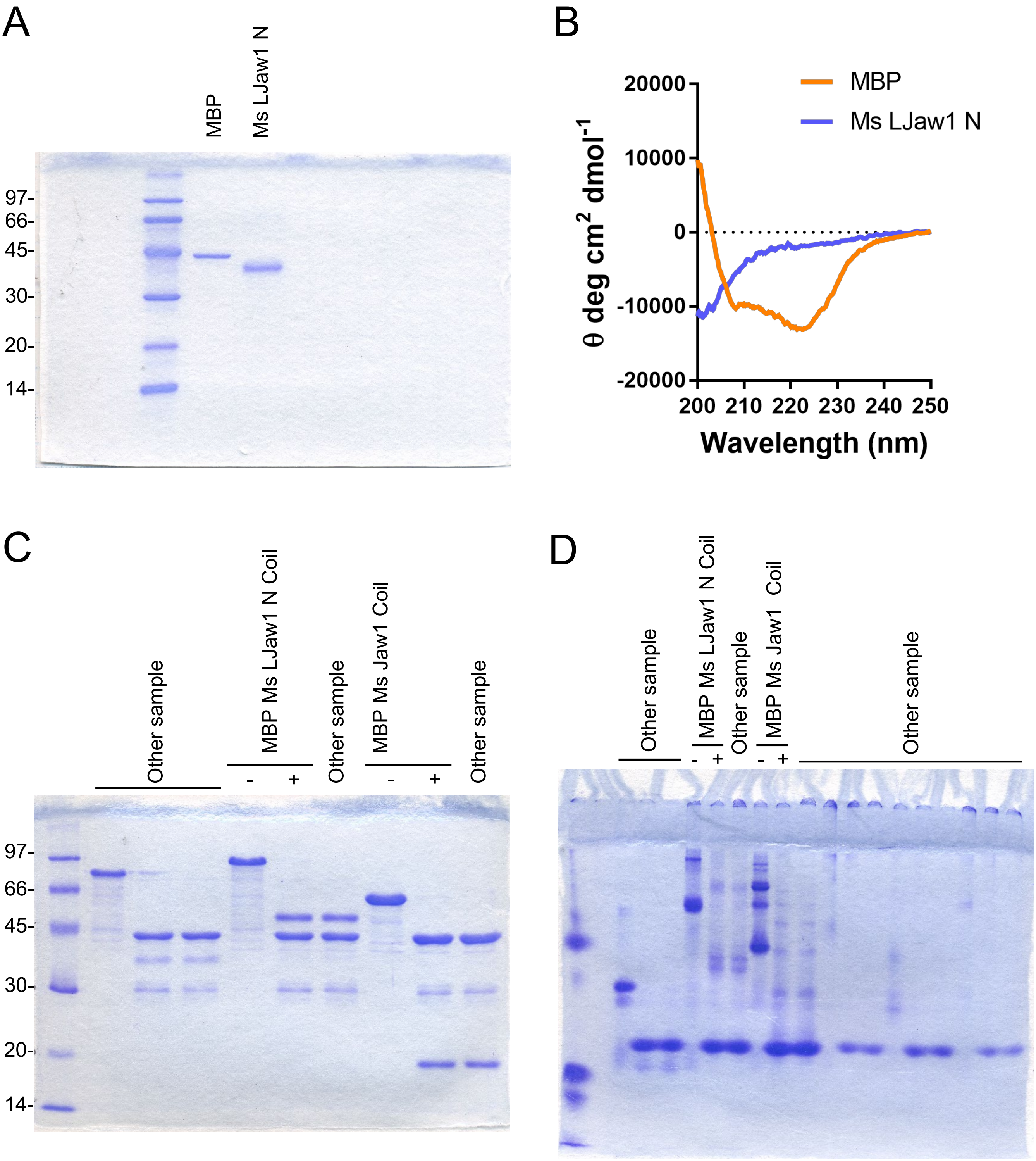

Supplementary Figure S5. Investigation of the function of Jaw1 N-terminal region on its oligomerization as an IDR. **A**) MBP Ms LJaw1 N was expressed in *E. coli* and purified using amylose resin. After the digestion of MBP tag with TEV protease, MBP and Ms LJaw1 N were separated by anion exchange chromatography and affinity chromatography using amylose resin and subjected to SDS-PAGE followed by CBB staining. **B**) The CD spectra of the purified MBP (orange) or Ms LJaw1 N (blue). **C, D**) The full-length gels of Fig. 4E and F are shown in (C) and (D), respectively.

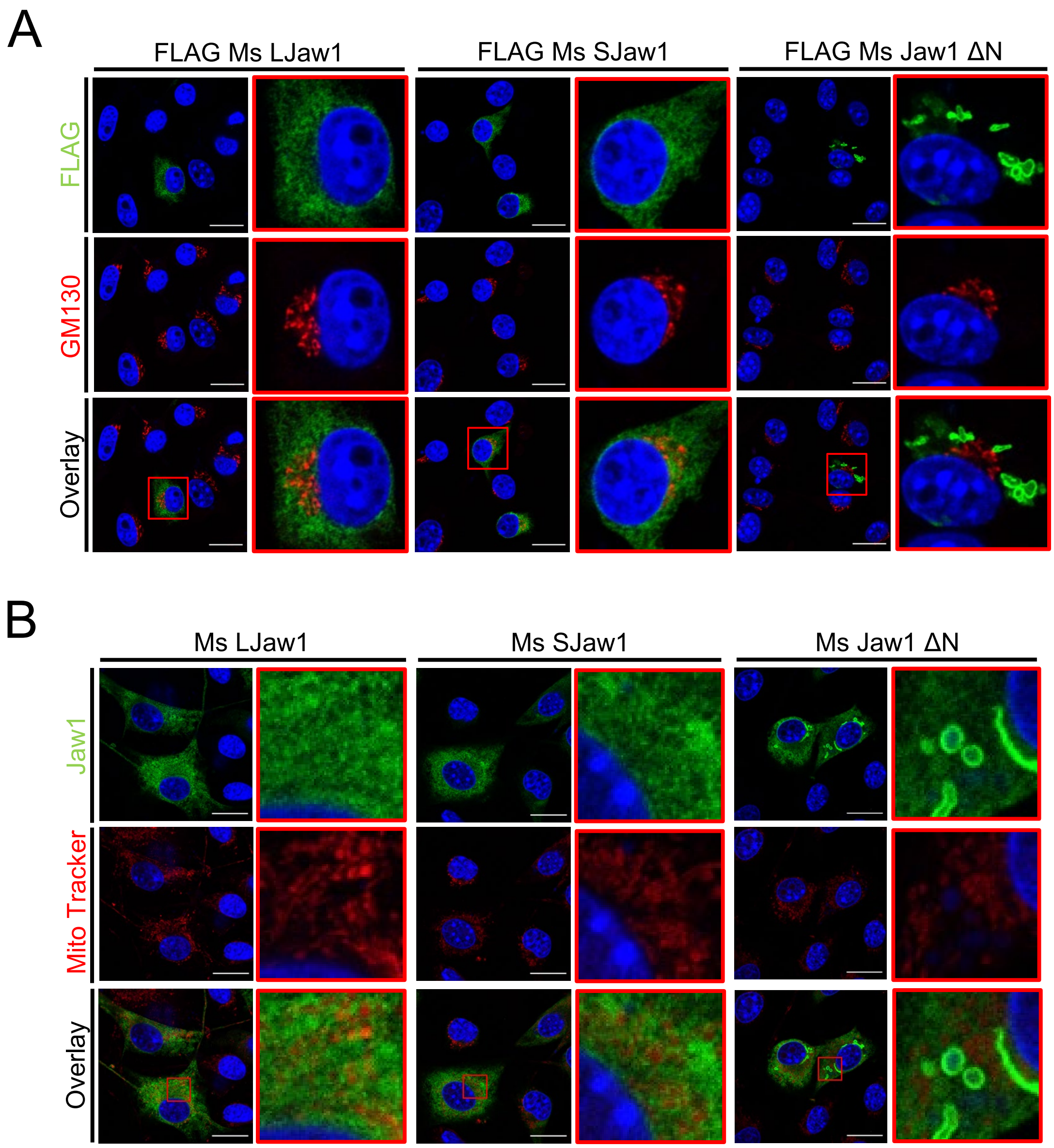

Supplementary Figure S6. Evaluation of golgi morphology and mitochondria under OSER formation. **A**) FLAG Ms LJaw1, FLAG Ms SJaw1 or FLAG Jaw1 ΔN were expressed in B16F10 cells by transfection. After incubation for 24 h, immunostaining was performed using an anti-FLAG rabbit antibody and an anti-GM130 mouse antibody as primary antibodies and an Alexa Fluor 488-labelled goat anti-rabbit IgG antibody and an Alexa Fluor 568-labelled goat anti-mouse IgG antibody as secondary antibodies. **B**) Ms LJaw1, Ms SJaw1 or Ms Jaw1 ΔN were expressed in B16F10 cells by transfection. After incubation for 24 h, the cells were treated with Mito Tracker, fixed and permeabilized followed by immunostaining using an anti-Jaw1 rat antibody as a primary antibody and an Alexa Fluor 488-labelled goat anti-rat IgG antibody as a secondary antibody. Nuclei were stained with Hoechst33342 (blue). The images were acquired by confocal microscopy. Scale bar; 20 µm. The magnified images corresponding to the area surrounded with red lines in each image were generated.

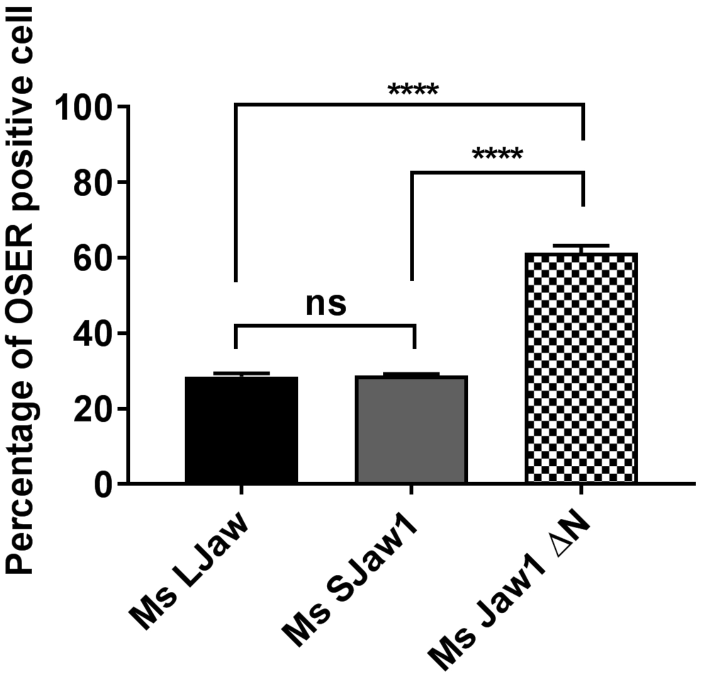

Supplementary Figure S7. Evaluation of the effects under OSER on the localization of IP_3R3. Counting of the cells having OSER structures out of the $Jaw1^+$ IP_3R3^+ cells in Fig. 6A (n =100). In the graph, the percentage of cells with OSER structures is shown based on the average of four independent experiments per condition. Error bars show the S. D. “ns”, not significant; ****, $P <$ 0.0001, Turkey Kramer’s t-test.

```
Human MESTPFSGVANQIHTLCERPTYGEVKDGALDVKRQHKCPGPTSGPSPGTNLSGCIRMNDD 60
Mouse -------------MLCVKGPPEQEPEDGALDVTRGCQCPLPTEGSILGQELLDCTRMNED 47
                         *   *  ****** *   ** **     *  *  * *** *

Human PSMEENGVERVCPESLLQSREYSSLPLPRHTSSTDGTITSSDPGLEILNMASCDLDRNSL 120
Mouse QSTDENGADHLYSESPSQLREYLTQPSSEQTSSSESTVTSSESGSDILHMASGDLDCKPL 107
       *  ***      **  * ***   *    ***   * ***  *  ** *** ***   *

Human CKKEEDTRSASPTIEAQGTSPAHDNIAFQDSTSKDKTILNLEAKEEPETIEEHKKEHASG 180
Mouse CEKEEEARAAS---AMQGTSLAPAAYGDYTSVGVAKAASQLEAGEELRTTENGGKGSAPG 164
      * ***  * **     **** *        *    *    *** **  * *   *  * *

Human DSVVSPLPVTTVKSVNVRQSENTSANEKEVEAEFLRLSLGFKCDWFTLEKRVKLEERSRD 240
Mouse ETEISMPPKASVKLVNFQQSENTSANEKEVEAEFLRLSLGLKCDWFTLEKRVKLEERSRD 224
          *  *   ** **  ********************** *******************

Human LAEENLKKEITNCLKLLESLTPLCEDDNQAQEIIKKLEKSIKFLSQCAARVASRAEMLGA 300
Mouse LAEENLKKEITNCLKLLESLTPLCEEDNQAQEIVKKLEKSIVLLSQCTARVASRAEMLGA 284
      ************************* ******* *******  **** ************

Human INQESRVSKAVEVMIQHVENLKRMYAKEHAELEELKQVLLQNERSFNPLEDDDDCQIKKR 360
Mouse INQESRVSRAVEVMIQHVENLKRMYAKEHAELEDLKQALLQNDRSFNSLPDEDDCQIKKR 344
      ******** ************************ *** **** **** * * ********

Human SASLNSKPSSLRRVTIASLPRNIGNAGMVAGMENNDRFSRRSSSWRILGSKQSEHRPSLP 420
Mouse SSSLNSKPSSLRRVTIASLPRNLGNVGLVSGMENNDRFSRRSSSWRILGTKQGEHRPSLH 404
      * ******************** ** * * ******************* ** ******

Human RFISTYSWADAEEEKCELKTKDDSEPSGEETVERTRKPSLSEKKNNPSKWDVSSVYDTIA 480
Mouse RFISTYSWADAEDERSDVKARDAPEPQGEEAVEGTRKPSLSERRSSTLAWDRGTICSSVA 464
      ************ *    *  *  ** *** ** ********       **        *

Human SWATNLKSSIRKANKALWLSIAFIVLFAALMSFLTGQLFQKSVDAAPTQQEDSWTSLEHI 540
Mouse SWVTHLQASFRRANRALWLTGLIIILIAALMSFLTGQLFQTAVEAAPTQEGDSWLSLEHI 524
      ** * *  * * ** ****    * * *************  * *****  *** *****

Human LWPFTRLRHNGPPPV 555
Mouse LWPFTRLGHDGPLPV 539
      ******* * ** **
```

Supplementary Figure S8. Comparison of the amino acid sequence between human and mouse LJaw1. Asterisk shows the conserved amino acids. Blue shaded; coiled-coil domain, gray shaded; trans-membrane domain, Yellow shaded; candidate mutation site in type I diabetes.

The percentage identity of amino acid sequences in each region between human and mouse Jaw1

Region	Percentage Identity (Identical amino acids/total amino acids)
**N-terminal region**	43.6% (61/140)
**coiled-coil domain**	92.9% (130/140)
**Stem region**	71.9% (97/135)
**Transmembrane domain**	75.0% (18/24)
**Luminal region**	75.0% (27/36)

Supplementary Table S1. The percentage identity of amino acid sequences in each region between human and mouse Jaw1. The numbers and percentage of identical amino acids in each region of human Jaw1 with that of mouse Jaw1 are shown.

The composition of amino acids in each region of human and mouse Jaw1

Human Jaw1	The percentage of amino acids			
	Hydrophobic	Neutral	Acidic	Basic
**N-terminal region**	27.27	41.26	18.88	12.59
**coiled-coil domain**	41.43	22.86	17.86	17.86
**Stem region**	23.70	42.22	16.30	17.78
**Transmembrane domain**	75.00	25.00	0	0
**Luminal region**	25.00	50.00	11.11	13.89

Mouse Jaw1	The percentage of amino acids			
	Hydrophobic	Neutral	Acidic	Basic
**N-terminal region**	27.86	47.14	17.14	7.86
**coiled-coil domain**	41.43	23.57	17.86	17.14
**Stem region**	25.19	42.96	14.07	17.78
**Transmembrane domain**	70.83	29.17	0.00	0.00
**Luminal region**	33.33	44.44	13.89	8.33

Supplementary Table S2. The composition of amino acids in each region of human and mouse Jaw1. The percentage of each amino acids group are shown. The amino acids with a positive value in Hphod. /Kyte & Doolittle of ProtScale (ExPASy Bioinformatics Resource Portal) were selected as hydrophobic ones. Gray shaded; charged amino acids group. Neutral; the percentage of amino acids except hydrophobic and charged group. Upper table, human Jaw1; bottom one, mouse Jaw1.

The composition of amino acids in each region (N1-N3) of mouse Jaw1 N-terminal region

Mouse Jaw1	The percentage of amino acids			
	Hydrophobic	Neutral	Acidic	Basic
**N-terminal region (44-183)**	27.86	47.14	17.14	7.86
**N1 (44-96)**	15.09	58.49	20.75	5.66
**N2 (97-127)**	43.33	26.67	20.00	10.00
**N3 (128-183)**	31.58	47.37	12.28	8.77

Supplementary Table S3. The composition of amino acids in each region (N1-N3) of mouse Jaw1 N-terminal region. The percentages of different amino acids groups per region are shown. The amino acids with a positive value in Hphod. /Kyte & Doolittle of ProtScale (ExPASy Bioinformatics Resource Portal) were selected as hydrophobic ones. Gray shaded; charged amino acids group. Neutral; the percentage of amino acids except hydrophobic and charged group.
